# Mathematical modelling of hollow microneedle-mediated transdermal drug delivery

**DOI:** 10.1007/s13346-025-01801-3

**Published:** 2025-02-06

**Authors:** Neil Benbrook, Wenbo Zhan

**Affiliations:** School of Engineering, University of Aberdeen, Aberdeen, AB24 3UE UK

**Keywords:** Hollow microneedle, Transdermal drug delivery, Drug nanocarrier, Mathematical model, Drug transport

## Abstract

Hollow microneedles represent a promising approach for overcoming the protective barrier of the stratum corneum, facilitating direct drug infusion into viable skin tissue and thereby enhancing the efficacy of transdermal delivery. However, delivery outcomes across different skin layers and into the systemic circulation can vary substantially due to the diverse properties of drug delivery systems, clinical settings, and environmental factors. The optimal strategies for enhancing the efficiency of hollow microneedle-mediated transdermal drug delivery remain to be elucidated. This study employs mathematical modelling and a reconstructed skin model with realistic anatomical structures to investigate drug transport and accumulation across different skin layers and into the bloodstream under different delivery conditions. The modelling results reveal the crucial role of interstitial fluid flow in determining drug transport in this transdermal delivery. Delivery outcomes of each skin layer and blood exhibit distinct responses to changes in delivery conditions. Specifically, increasing the vascular permeability or nanocarrier diffusivity raises drug concentration in the blood or reticular dermis, respectively, while leading to reductions in other skin layers. The use of microneedles with narrower infusion channels can only enhance drug availability in the viable epidermis. Optimisation requires a tailored approach to several parameters depending on the target skin layer, including drug release rate, infusion rate, infusion duration, and microneedle length. Environmental factors that promote trans-epidermal water loss can increase drug concentration in the viable epidermis but have a limited impact on deeper skin tissues. The findings support the selection or customisation of hollow microneedles and nanocarriers to address specific therapeutic needs, such as targeting specific skin layers or systemic circulation, while minimising the risk of side effects from high drug concentrations in normal tissues. This study provides guidance for optimising transdermal drug delivery systems.

**Graphical Abstract:**

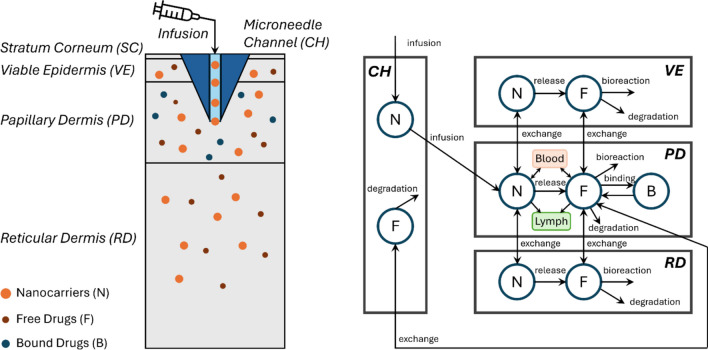

## Introduction

Transdermal drug delivery is a method of administering drugs through the skin for systemic distribution [1]. It is favoured in practice due to several advantages, such as improved patient compliance, less invasion and convenience to perform [2, 3]. Although this delivery method has been employed to manage various disorders, such as diabetes, Alzheimer's, and tumours, as well as injury [4, 5], there is still a large room for improvement in its efficacy. Subject to the anatomic structure of the skin, drugs applied to the skin surface must traverse the stratum corneum (SC) and viable epidermis (VE) to reach the papillary dermis (PD), where blood capillaries are located. These capillaries facilitate the transfer of drugs into the blood for systemic absorption, while the rest of the drugs in the PD can either enter the lymphatic system, whose vessels run parallel to blood capillaries [6] or penetrate deeper into the reticular dermis (RD). SC is a lipid-protein biphasic structure composed of layers of corneocytes sealed by densely packed lipids [7]. As the outermost layer of the skin, it acts as a protective barrier that is impermeable to a variety of external substances, including most drugs [8]. This presents a significant challenge for transdermal drug delivery, as overcoming this barrier is essential for successful medication absorption.

Hollow microneedles provide a promising solution to overcome this barrier [9]. With a hollow channel (CH) inside, these microneedles can effectively pierce through the SC to access the viable skin tissues beneath, allowing drugs to be infused directly into the skin tissue through the channel [10, 11]. Various hollow microneedle designs have been developed to achieve this goal [11]. However, delivery outcomes are still limited by the rapid elimination of small-molecule drugs in the body. To further improve treatment efficacy, nanocarriers loaded with small-molecule drugs are used for administration. Fabricated using materials that are inert to undesired bioreactions, these nanocarriers encapsulate the therapeutic compounds internally to reduce elimination, thereby providing a continuous drug supply [12]. The viability of the combination of hollow microneedles and nanocarriers has been demonstrated [13, 14].

Transdermal drug delivery involves multiple interconnected drug transport processes that are heavily affected by the dynamic properties of skin tissue [1, 15], which can vary significantly among patients [16–18]. This complexity makes it challenging to isolate individual influencing factors through in vivo experiments alone. In this context, mathematical modelling emerges as a valuable tool [19–21]. Since the models can predict the time-varying concentration of a drug and its spatial distribution, this approach is particularly advantageous for studying the transdermal transport of drugs across different skin layers. Using a group of governing equations to depict drug transport processes, Calcutt et al. conducted a numerical analysis to evaluate the effects of various factors both independently and in combination [22]. This numerical tool was also applied by Anissimov and Roberts to examine the effects of different properties of the subpapillary plexus on the transport of small-molecule drugs in viable skin tissues [23]. Machekposhti et al. simulated drug delivery to the skin using polymer microneedles, with their modelling predictions showing strong agreement with experimental data [24]. Newell and Zhan numerically evaluated the delivery outcomes of hydrogel microneedles and solid microneedles coupled with medicated adhesive patches [25, 26]. However, the impact of various factors related to drug delivery systems and their operation on hollow microneedle-mediated transdermal delivery remains unclear, hindering the optimisation of this delivery method.

In this study, transdermal drug delivery using hollow microneedles is mathematically modelled under different delivery conditions to examine the role of multiple properties of hollow microneedles and nanocarriers, clinical settings and environmental factors. The model is constructed to encompass the fundamental drug transport mechanisms, including drug transport among skin layers by diffusion and with infusate and interstitial fluid flow, drug release, blood and lymphatic drainage, and enzyme-involved and non-enzyme-involved reactions. Delivery outcomes in each skin layer and blood are assessed through drug exposure which is derived from the predicted local drug concentrations.

## Materials and methods

### Mathematical models

The model comprises governing equations describing the transfer of infusate (IFS), interstitial fluid (ISF) and drugs within and among the microneedle channel, skin tissues, and blood and lymphatic circulating system. While water molecules can penetrate the SC, the transport mechanism differs greatly from that in viable skin tissues [27]. Since this study predominantly concentrates on viable skin tissues, water flux through the SC is considered with regard to trans-epidermal water loss (TEWL). The skin tissue is assumed to be homogeneous, disregarding variations in tissue properties among individuals and deformation induced by microneedling.

#### Fluid transport model

Infusate is administrated into the skin through the microneedle channel. Viable skin tissues, including VE, PD and RD, can be regarded as porous media because of their tissue microstructures where ISF travels in the gaps between cells. Therefore, the flow of IFS and ISF can be depicted by the continuity equation and momentum conservation equation, as:

1 $$\nabla \cdot {\mathbf{v}}_{\text{IFS}}=\begin{array}{cc}0,& \text{in CH}\end{array}$$

2 $${\rho }_{\text{IFS}}\frac{\partial {\mathbf{v}}_{\text{IFS}}}{\partial t}+{\rho }_{\text{IFS}}\left({\mathbf{v}}_{\text{IFS}}\cdot \nabla {\mathbf{v}}_{\text{IFS}}\right)=\begin{array}{cc}-\nabla {p}_{\text{IFS}}+{\mu }_{\text{IFS}}{\nabla }^{2}{\mathbf{v}}_{\text{IFS}},& \text{in CH}\end{array}$$

where $\mathbf{v}$ and $p$ are the flow velocity and pressure, respectively. $\rho$ is the density of the fluid, and $\mu$ is its dynamics viscosity.

3 $$\nabla \cdot {\mathbf{v}}_{\text{ISF}}=\left\{\begin{array}{cc}{R}_{\text{BL}}-{R}_{\text{LY}}, & \text{in PD}\\ 0, & \text{in VE and RD}\end{array}\right.$$

4 $${\rho }_{\text{ISF}}\frac{\partial {\mathbf{v}}_{\text{ISF}}}{\partial t}+{\rho }_{\text{ISF}}\left({\mathbf{v}}_{\text{ISF}}\cdot \nabla {\mathbf{v}}_{\text{ISF}}\right)=\begin{array}{cc}-\nabla {p}_{\text{ISF}}+{\mu }_{\text{ISF}}{\nabla }^{2}{\mathbf{v}}_{\text{ISF}}-\frac{{\mu }_{\text{ISF}}}{{\kappa }_{\text{TIS}}}{\mathbf{v}}_{\text{ISF}},& \text{in VE},\text{PD and RD}\end{array}$$

where $\kappa$ is the tissue permeability which describes the ability of the tissue to enable the passage of ISF. The rates of fluid exchange between the PD and the blood (${R}_{\text{BL}}$) and the lymph (${R}_{\text{LY}}$) are controlled by Starling’s law, as:

5 $${R}_{\text{BL}}={L}_{\text{BL}}\frac{{S}_{\text{BL}}}{{V}_{\text{TIS}}}\left[{p}_{\text{BL}}-{p}_{\text{ISF}}-{\sigma }_{\text{T}}\left({\pi }_{\text{BL}}-{\pi }_{\text{ISF}}\right)\right]$$

6 $${R}_{\text{LY}}={L}_{\text{LY}}\frac{{S}_{\text{LY}}}{{V}_{\text{TIS}}}\left({p}_{\text{ISF}}-{p}_{\text{LY}}\right)$$

in which $L$ is the hydraulic conductivity of the vessel wall, and $S$ is its surface area. $V$ is the local tissue volume. ${\sigma }_{\text{T}}$ stands for the osmotic reflection coefficient. $\pi$ is the osmotic pressure. The subscripts of BL and LY refer to the blood and lymph, respectively, and TIS is for the skin tissue.

#### Drug transfer model

**Fig. 1:**
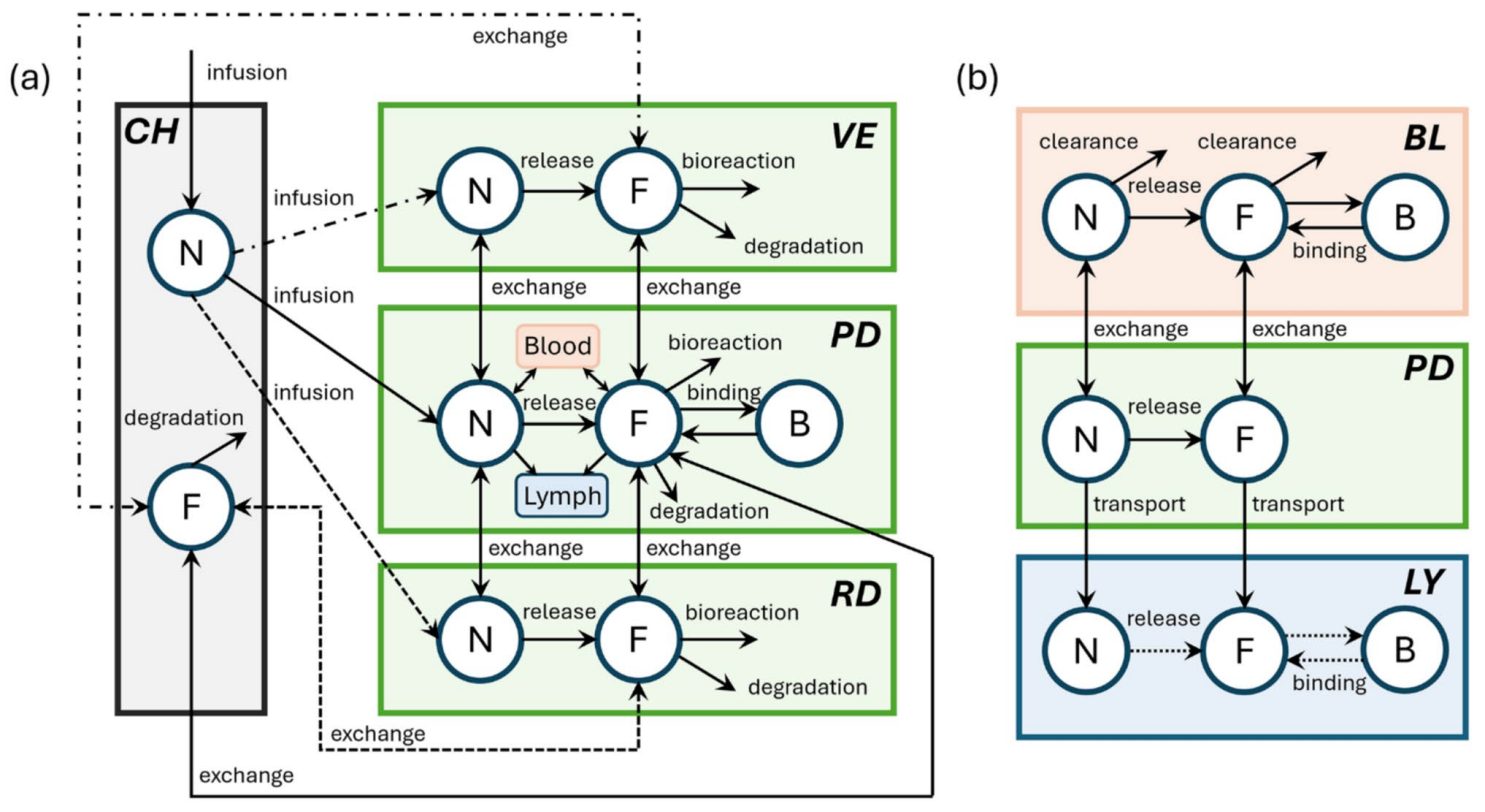
Schematical diagram of hollow microneedle-mediated transdermal drug delivery. The overview of drug nanocarrier delivered to skin tissues using hollow microneedles **a**, and a close observation of drug transport between the PD, BL and LY. The lymphatic system is considered a sink for drug nanocarriers and released drugs **b**. Hence, the drug transport within the lymphatic system is not modelled explicitly, marked by the dotted line. The infusion through the channel is subject to the insertion depth of microneedles into the skin. The infusion occurs at the PD in the baseline delivery, marked by the solid line. With the variation of the microneedle length, the infusion site can also be located in the VE or RD, marked by the dash-dotted line and dashed line, respectively

Figure 1 presents the transport of drugs in different forms among the microneedle channel, skin, blood and lymphatic circulating systems. The letters N, F and B refer to drug nanocarriers, released drugs in the free form, and drugs in the bound form to protein, respectively. As shown in Fig. 1(a), drugs are administered into the skin tissues via the microneedle channel in a nanocarrier-encapsulated form, with the assumption that no drug release occurs prior to entering the tissue. Once within the skin, the nanocarriers commence the release of the loaded drugs. The residual nanocarriers and the released drugs can transfer within the skin layer where the microneedle tip is located and subsequently spread to adjacent skin layers. Notably, both nanocarriers and free drugs are capable of bidirectional transfer between different skin layers and are subject to elimination due to bioreactions and physical degradation in all layers. Of particular significance is the presence of the papillary plexus in the PD, where proteins extravasated from the bloodstream can bind to free drugs. Furthermore, nanocarriers and free drugs may enter the blood and lymphatic circulatory systems at this layer. A close view of the drug transport processes between PD, BL and LY is illustrated in Fig. 1(b). Nanocarriers within the circulatory systems can continue to release their payload, while free drugs can bind to proteins. Both nanocarriers and free drugs present in the bloodstream are also subject to clearance by various organs, such as the kidneys and liver. Since this study is focused on transdermal delivery to skin tissues and the blood, the lymphatic system serves as a sink for drug nanocarriers and released drugs in the model.

##### Drug transfer in the microneedle channel

The transfer of drug nanocarriers in the microneedle channel depends on convection with infusate flow and diffusion. All small-molecule drugs are assumed to be well encapsulated in the nanocarriers before entering the skin tissues. The nanocarrier concentration in the microneedle channel (${C}_{\text{N},\text{CH}}$) can be calculated by:

7 $$\frac{\partial {C}_\text{N,CH}}{\partial t}=\nabla \cdot \left({D}_\text{N,CH}\nabla {C}_\text{N,CH}\right)-\nabla \cdot \left({{\mathbf{v}}}_{{\text{IFS}}}{C}_\text{N,CH}\right)$$

where ${D}_{\text{N},\text{CH}}$ is the diffusion coefficient of nanocarriers in the channel. The local concentration of free drugs (${C}_{\text{F},\text{CH}}$) depends on diffusion and convection with infusate flow, and drug degradation, as:

8 $$\frac{\partial {C}_{\text{F},\text{CH}}}{\partial t}=\nabla \cdot \left({D}_{\text{F},\text{CH}}\nabla {C}_{\text{F},\text{CH}}\right)-\nabla \cdot \left({\mathbf{v}}_{\text{IFS}}{C}_{\text{F},\text{CH}}\right)-{k}_{\text{d},\text{CH}}{C}_{\text{F},\text{CH}}$$

where ${D}_{\text{F},\text{CH}}$ is the diffusion coefficient of free drugs in the channel. ${k}_{\text{d},\text{CH}}$ is the physical degradation rate of free drugs.

##### Drug transfer in the viable epidermis

Nanocarriers travel via diffusion and convection with ISF flow in the VE. The concentration (${C}_{\text{N},\text{VE}}$) is also affected by drug release, as:

9 $$\frac{\partial {C}_\text{N,VE}}{\partial t}=\nabla \cdot \left({D}_\text{N,VE}\nabla {C}_\text{N,VE}\right)-\nabla \cdot \left({{\mathbf{v}}}_{{\text{ISF}}}{C}_\text{N,VE}\right)-{k}_\text{rel,VE}{C}_\text{N,VE}$$

in which ${D}_\text{N,VE}$ is the local nanocarrier diffusion coefficient. ${k}_\text{rel,VE}$ is the release rate of drugs from nanocarriers in the VE. The free drug concentration (${C}_\text{F,VE}$) depends on the drug diffusive transport, convective transport, bioreactions, and physical degradation. The drug release from nanocarriers serves as a source of free drugs in this layer. This concentration can then be calculated using:

10 $$\frac{\partial {C}_\text{F,VE}}{\partial t}=\nabla \cdot \left({D}_\text{F,VE}\nabla {C}_\text{F,VE}\right)-\nabla \cdot \left({{\mathbf{v}}}_{{\text{ISF}}}{C}_\text{F,VE}\right)+{k}_\text{rel,VE}{C}_\text{N,VE}-\frac{{V}_\text{max}{C}_\text{F,VE}}{{v}_\text{m}+{C}_\text{F,VE}}-{k}_\text{d,VE}{C}_\text{F,VE}$$

in which ${D}_\text{F,VE}$ and ${k}_\text{d,VE}$ are the local diffusion coefficient and physical degradation rate of free drugs, respectively. ${V}_\text{max}$ and ${v}_\text{m}$ are the Michaelis–Menten kinetics constants for the drug bioreactions.

##### Drug transfer in the papillary dermis

The transfer of nanocarriers in the PD is not only dependent on diffusion, convection and drug release but also determined by the exchange between the tissue and circulating systems. The concentration (${C}_{\text{N},\text{PD}}$) is governed by:

11 $$\frac{\partial {C}_\text{N,PD}}{\partial t}=\nabla \cdot \left({D}_\text{N,PD}\nabla {C}_\text{N,PD}\right)-\nabla \cdot \left({{\mathbf{v}}}_{{\text{ISF}}}{C}_\text{N,PD}\right)-Ex\left({C}_\text{N,PD},{C}_\text{N,BL}\right)- {R}_\text{LY}{C}_\text{N,PD}-{k}_\text{rel,PD}{C}_\text{N,PD}$$

where ${D}_\text{N,PD}$ is the nanocarrier diffusion coefficient in the PD, and ${k}_\text{rel,PD}$ is the drug release rate. The nanocarrier exchange rate between the PD and the BL, $Ex\left({C}_\text{N,PD},{C}_\text{N,BL}\right)$, is defined as:

12 $$Ex\left({C}_{\text{N},\text{PD}},{C}_{\text{N},\text{BL}}\right)={P}_{\text{N}}\frac{{S}_{\text{BL}}}{{V}_{\text{TIS}}}\left({C}_{\text{N},\text{PD}}-{C}_{\text{N},\text{BL}}\right)\frac{{\text{Pe}}_{\text{tb},\text{N}}}{{e}^{{\text{Pe}}_{\text{tb},\text{N}}}-1}-{R}_{\text{BL}}\left(1-{\sigma }_{\text{N}}\right){C}_{\text{N},\text{BL}}$$

where ${C}_{\text{N},\text{BL}}$ is the concentration of drug nanocarriers in the BL. ${P}_{\text{N}}$ is the vascular permeability of nanocarriers. ${\sigma }_{\text{N}}$ is the osmotic reflection coefficient of nanocarriers. ${\text{Pe}}_{\text{tb},\text{N}}$ is the Péclet number of nanocarriers, as:

13 $${\text{Pe}}_{\text{tb},\text{N}}=\frac{{R}_{\text{BL}}\left(1-{\sigma }_{\text{N}}\right)}{{P}_{\text{N}}{S}_{\text{BL}}/{V}_{\text{TIS}}}$$

Besides the mechanisms including diffusion, convection, drug release, physical degradation, and bioreaction, the free drug concentration in the PD (${C}_{\text{F},\text{PD}}$) also depends on the drug exchange between the PD and the circulating systems, and binding to protein, as:

14 $$\frac{\partial {C}_{\text{F},\text{PD}}}{\partial t}=\nabla \cdot \left({D}_{\text{F},\text{PD}}\nabla {C}_{\text{F},\text{PD}}\right)-\nabla \cdot \left({\mathbf{v}}_{\text{ISF}}{C}_{\text{F},\text{PD}}\right)+{k}_{\text{rel},\text{PD}}{C}_{\text{N},\text{PD}}-{k}_{\text{d},\text{PD}}{C}_{\text{F},\text{PD}}-\frac{{V}_{\text{max}}{C}_{\text{F},\text{PD}}}{{v}_{\text{m}}+{C}_{\text{F},\text{PD}}}-Ex\left({C}_{\text{F},\text{PD}},{C}_{\text{F},\text{BL}}\right)-{R}_{\text{LY}}{C}_{\text{F},\text{PD}}-\left({k}_{\text{pb}}{C}_{\text{F},\text{PD}}-{k}_{\text{pu}}{C}_{\text{B},\text{PD}}\right)$$

where ${D}_{\text{F},\text{PD}}$ and ${k}_{\text{d},\text{PD}}$ are the diffusion coefficient and physical degradation of free drugs in the PD, respectively. $Ex\left({C}_{\text{F},\text{PD}},{C}_{\text{F},\text{BL}}\right)$ presents the exchange rate of free drugs between the PD and the BL, which has the same definition in Eqs. (12) and (13) using the concentration and properties of free drugs. Drug binding is a two-way process, for which ${k}_{\text{pb}}$ is the association rate of free drugs with protein and ${k}_{\text{pu}}$ is the dissociation rate. The concentration of bound drugs in this skin layer (${C}_{\text{B},\text{PD}}$) is controlled by:

15 $$\frac{d{C}_{\text{B},\text{PD}}}{dt}={k}_{\text{pb}}{C}_{\text{F},\text{PD}}-{k}_{\text{pu}}{C}_{\text{B},\text{PD}}$$

##### Drug transfer in the reticular dermis

Nanocarriers mainly travel by convection and diffusion in the RD. The local drug release can also affect their concentration (${C}_{\text{N},\text{RD}}$), as:

16 $$\frac{\partial {C}_\text{N,RD}}{\partial t}=\nabla \cdot \left({D}_\text{N,RD}\nabla {C}_\text{N,RD}\right)-\nabla \cdot \left({{\mathbf{v}}}_{{\text{ISF}}}{C}_\text{N,RD}\right)-{k}_\text{rel,RD}{C}_\text{N,RD}$$

where ${D}_\text{N,RD}$ is the diffusion coefficient of nanocarriers in the RD, and ${k}_\text{rel,RD}$ is the drug release rate. The local free drug concentration (${D}_\text{F,RD}$) is dependent on convective and diffusive transport, drug release, bioreaction, and physical degradation, as:

17 $$\frac{\partial {C}_\text{F,RD}}{\partial t}=\nabla \cdot \left({D}_\text{F,RD}\nabla {C}_\text{F,RD}\right)-\nabla \cdot \left({{\mathbf{v}}}_{{\text{ISF}}}{C}_\text{F,RD}\right)+{k}_\text{rel,RD}{C}_\text{N,RD}-\frac{{V}_\text{max}{C}_\text{F,RD}}{{v}_\text{m}+{C}_\text{F,RD}}-{k}_\text{d,RD}{C}_\text{F,RD}$$

in which ${D}_\text{F,RD}$ is the diffusion coefficient of free drugs. ${k}_\text{d,RD}$ is their physical degradation in this layer.

##### Drug transfer in the blood

The blood concentration of drug nanocarriers (${C}_{\text{N},\text{BL}}$) relies on the exchange between the BL and the PD, local drug release, and plasma clearance, as:

18 $$\frac{d{C}_\text{N,BL}}{dt}=\frac{{V}_\text{PD}N}{{V}_\text{dis,N}}Ex\left({C}_\text{N,BL},{C}_\text{N,PD}\right)-{k}_\text{rel,BL}{C}_\text{N,BL}-{k}_\text{clr,N}{C}_\text{N,BL}$$

where ${V}_\text{PD}$ is the volume of PD, and $N$ is the number of microneedles in a microneedle array. ${V}_\text{dis,N}$ is the distribution volume of nanocarriers. ${k}_\text{rel,BL}$ and ${k}_\text{clr,N}$ are the local drug release rate and plasma clearance rate, respectively.

The free drug concentration in the BL (${C}_{\text{F},\text{BL}}$) depends on the exchange with the PD, local drug release, binding with protein, and plasma clearance, as:

19 $$\frac{d{C}_\text{F,BL}}{dt}=\frac{{V}_\text{PD}N}{{V}_\text{dis,F}}Ex\left({C}_\text{F,BL},{C}_\text{F,PD}\right)+{k}_\text{rel,BL}{C}_\text{N,BL}-\left({k}_\text{pb}{C}_\text{F,BL}-{k}_\text{pu}{C}_\text{B,BL}\right)-{k}_\text{clr,F}{C}_\text{F,BL}$$

where ${V}_\text{dis,F}$ is the distribution volume of free drugs. ${k}_\text{clr,F}$ is the rate of plasma clearance. The concentration of bound drugs in the BL (${C}_\text{B,BL}$) is defined as:

20 $$\frac{d{C}_\text{B,BL}}{dt}={k}_\text{pb}{C}_\text{F,BL}-{k}_\text{pu}{C}_\text{B,BL}$$

### Model geometry

**Fig. 2:**
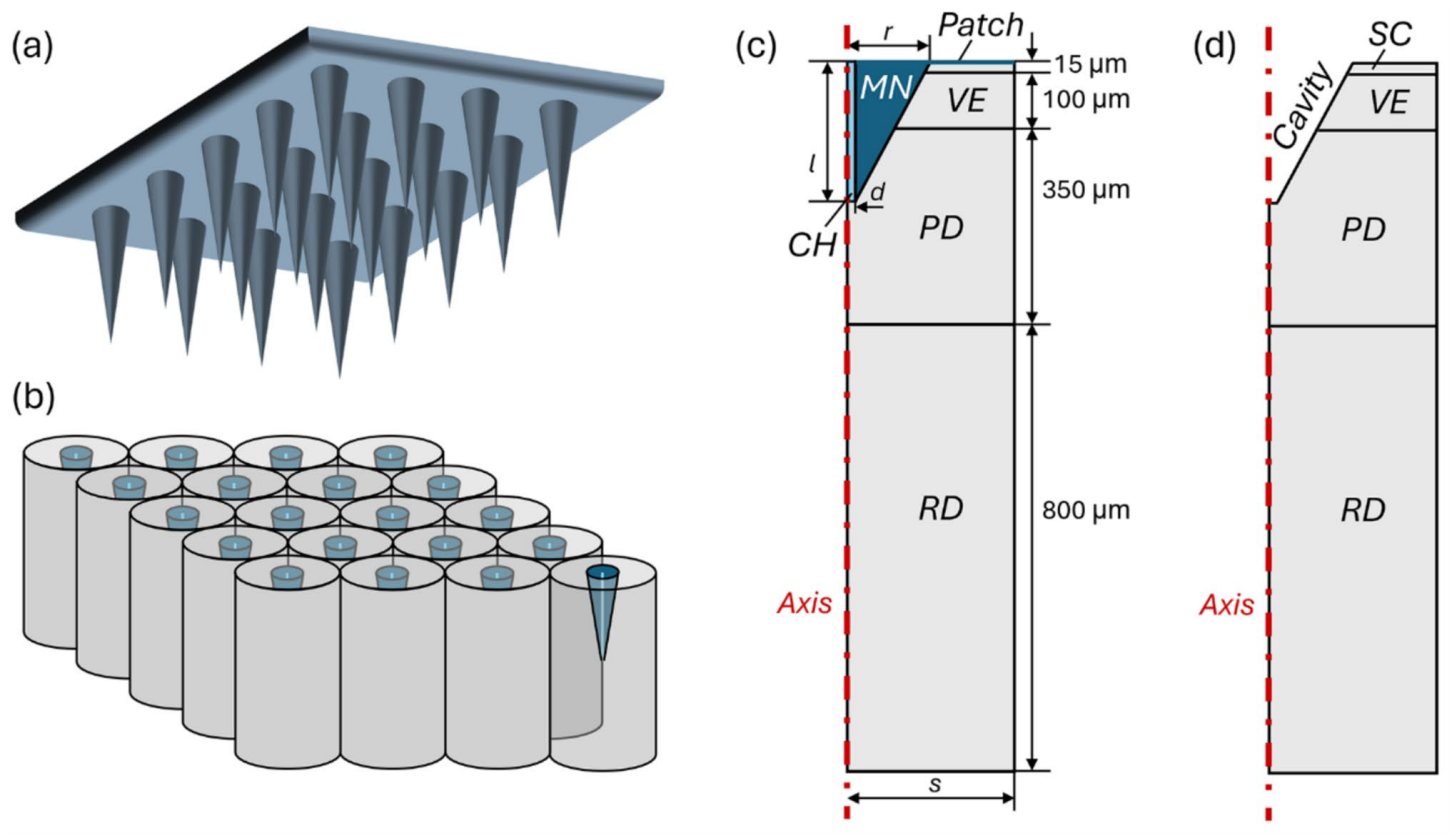
Model geometry. Schematical sketch of microneedle array **a**, arrangement of representative elementary volumes **b**, computational domain for the transdermal delivery during drug infusion **c**, and after microneedle removal **d**. The skin tissues, microneedle body and channel are marked in light grey, dark blue and light blue, respectively. $l$ is the microneedle length, $d$ is the half of channel width, $r$ is the microneedle base radius and $s$ is half of the tip-to-tip distance between two neighbouring microneedles. The patch of microneedles that covers the skin surface is impermeable, preventing mass loss to the environment during infusion. Therefore, its thickness is not considered explicitly in the simulations. The microneedle wall and tip become the surface of the cavity after the removal of microneedles

Given that multiple microneedles are commonly equally spaced on a supporting patch, a representative elementary volume (REV) can be identified, as illustrated in Fig. 2. The mathematical model describing the hollow microneedle-mediated transdermal drug delivery is solved in 2D axisymmetric configurations for the delivery during infusion and after microneedle removal. These geometrical models incorporate realistic thicknesses of the skin layers: $15 \upmu{\text{m}}$ for SC, $100 \upmu{\text{m}}$, for VE, and $350 \upmu{\text{m}}$ for PD [22, 28]. The RD which is $800 \upmu{\text{m}}$ thick is positioned midway between the papillary plexus and the reticular plexus [22]. Microneedle geometrical properties vary significantly based on design and fabrication methods [29, 30]. In the baseline study, we employ a configuration of $10\times 10$ cone-shaped microneedles spaced $600 \upmu{\text{m}}$ apart [31], each with a microneedle length and channel width of $250 \upmu{\text{m}}$ and $30 \upmu{\text{m}}$, respectively. The cylindrical infusion channel is positioned at the centre of the microneedle [32]. The radius of the microneedle at its basis is $150 \upmu{\text{m}}$. These dimensions enable the microneedles to reach the PD which embeds blood capillaries; further analyses of geometrical properties are outlined in subsequent studies. The computational mesh comprises around $\text{70,000}$ triangular elements, which have been validated through a mesh quality test. The finest elements of $0.005 \upmu{\text{m}}$ in size are utilised at the microneedle tip and the interfaces between the microneedle and skin tissues for precise predictions.

### Model parameters

The properties of skin tissues and drugs in different forms are considered constant which are independent of time. Doxorubicin is selected as the representative drug in this study [33, 34]. Tables 1 and 2 summarise the baseline values of these parameters for tissues and drugs, respectively. It is reported that the cavity could heal within 2 h after the microneedle was removed [30, 35]. However, further investigations have revealed that the lifetime of the cavity may be prolonged in elderly individuals, with experimental results suggesting that healing may take 24 h or even longer [36]. Therefore, this delivery using hollow microneedles is simulated in a timeframe of 24 h in this study. The justifications for key model parameters are given below.

**Table 1:** Model parameters for skin tissue properties

Symbol	Parameter	Unit	CH	VE	PD	RD
${\kappa }_{\text{TIS}}$	Tissue permeability	m^2^	$-$	$1.0\times {10}^{-16}$ [37]	$1.0\times {10}^{-16}$ [37]	$1.0\times {10}^{-16}$ [37]
${\mu }_{\text{ISF}}$	ISF viscosity	mPas	$-$	$0.78$ [38]	$0.78$ [38]	$0.78$ [38]
${\mu }_{\text{IFS}}$	IFS Viscosity	mPas	$0.78$ [39]	$-$	$-$	$-$
${\rho }_{\text{ISF}}$	ISF density	kgm^−3^	$-$	$1000$ [40]	$1000$ [40]	$1000$ [40]
${\rho }_{\text{IFS}}$	IFS density	kgm^−3^	1000 [39]	$-$	$-$	$-$
${\pi }_{\text{BL}}$	Osmotic pressure of blood	Pa	$-$	$-$	$2670$ [41]	$-$
${\pi }_{\text{ISF}}$	ISF osmotic pressure	Pa	$-$	$-$	$1330$ [41]	$-$
${\sigma }_{\text{T}}$	Osmotic reflection coefficient for proteins in the blood	$-$	$-$	$-$	$0.91$ [41]	$-$
${p}_{\text{BL}}$	Intracapillary pressure	Pa	$-$	$-$	$2080$ [41]	$-$
${L}_{\text{BL}}$	Hydraulic conductivity of the blood vessel wall	mPa^−1^ s^−1^	$-$	$-$	$2.7\times {10}^{-12}$ [41]	$-$
${S}_{\text{BL}}/{V}_{\text{TIS}}$	Area of capillary surface per tissue volume	m^−1^	$-$	$-$	$6.0\times {10}^{3}$ [42]	$-$
${{L}_{\text{LY}}S}_{\text{LY}}/{V}_{\text{TIS}}$	Rate of ISF loss to lymphatics	Pa^−1^ s^−1^	$-$	$-$	$4.2\times {10}^{-7}$ [41]	$-$
${p}_{\text{LY}}$	Intra-lymphatic pressure	Pa	$-$	$-$	$0$ [41]	$-$

**Table 2:** Model parameters for drug properties

Symbol	Parameter	Unit	Drug nanocarrier	Smal-molecule drug
${D}_{\text{CH}}$	Diffusion coefficient in microneedle CH	m^2^s^−1^	$2.9\times {10}^{-12}$ [43]	$3.8\times {10}^{-10}$ [44]
${D}_{\text{TIS}}$	Diffusion coefficient in viable skin tissues	m^2^s^−1^	$1.0\times {10}^{-13}$ [37]	$1.0\times {10}^{-10}$ [37]
${k}_{\text{rel}}$	Drug release rate	s^−1^	$1.0\times {10}^{-4}$ [45]	$-$
${k}_{\text{d}}$	Drug degradation rate	s^−1^	$-$	$5.6\times {10}^{-6}$ [46]
${V}_{\text{max}}$	Michaelis–Menten constant	molm^−3^ s^−1^	$-$	$0.512$ [47]
${v}_{\text{m}}$	Michaelis–Menten constant	molm^−3^	$-$	$6.7\times {10}^{-3}$ [47]
${k}_{\text{bp}}$	Drug association rate with protein	s^−1^	$-$	$0.833$ [48]
${k}_{\text{up}}$	Drug dissociation rate from protein	s^−1^	$-$	$0.278$ [48]
$\sigma$	Osmotic reflection coefficient	$-$	$1.0$ [49]	$0.15$ [38]
$P$	Vascular permeability	ms^−1^	$1.0\times {10}^{-9}$ [50]	$3.8\times {10}^{-7}$ [37]
${C}_{\text{in}}$	Administration dose	M	1.0 [51]	$-$
${k}_{\text{clr}}$	Rate of plasma clearance	s^−1^	$5.0\times {10}^{-5}$ [37]	$1.0\times {10}^{-4}$ [37]
${V}_{\text{dis}}$	Distribution volume	m^3^	$1.8\times {10}^{-2}$ [37]	$2.0\times {10}^{-2}$ [37]

#### Nanocarrier properties

Release rate (${k}_{\text{rel}}$) represents the time scale for nanocarriers to release the loaded small-molecule drugs, directly determining the therapeutic effects. Subject to a variety of factors, including the nanocarrier formulation and surrounding environment, its value can vary in a considerably large range. For instance, the release rate of a polymeric nanocarrier is reported to be $9.5\times {10}^{-5} {\text{s}}^{-1}$ [52]. Thermosensitive liposomes swiftly release their payload within seconds when the surrounding temperature surpasses the phase transition temperature of the lipid membrane; the release rate could reach $5.4\times {10}^{-2} {\text{s}}^{-1}$ [53]. Therefore, a large range from ${1.0\times 10}^{-5} {\text{s}}^{-1}$ to ${1.0\times 10}^{-1} {\text{s}}^{-1}$ is applied, and ${1.0\times 10}^{-4} {\text{s}}^{-1}$ is selected as the baseline.

Vascular permeability (${P}_{\text{N}}$) reflects the ability of nanoparticles to pass through the blood capillary wall. It assumes a pivotal role in dictating the systemic availability of drugs, consequently influencing the efficacy of transdermal delivery. Although capillary walls featuring tight intercellular clefts and continuous basement membranes are almost impermeable to large molecules [43], nanocarrier surfaces can be modified using certain ligands, such as transferrin, to facilitate their passage into the bloodstream. The measured vascular permeability of nanocarriers was located in a range from ${10}^{-11}\text{ m}/\text{s}$ to ${10}^{-9}\text{ m}/\text{s}$ through experiments [54, 55]. Theoretical analyses additionally demonstrated this rate could be elevated to the order of ${10}^{-7} \text{m}/\text{s}$ with a reduction in particle size to a few nanometres [56]. Therefore, $1.0\times {10}^{-11}\sim 1.0\times {10}^{-7} \text{m}/\text{s}$ is selected as the range of vascular permeability of nanocarriers; $1.0\times {10}^{-9}\text{ m}/\text{s}$ is the baseline value in Table 2.

Diffusion coefficient (${D}_{\text{N}}$) reflects the ability of nanocarriers to move in the skin owing to the thermal motion of molecules. It is influenced by various factors related to both the nanocarriers and transport medium, encompassing nanocarrier dimension and tissue compositions. For instance, the diffusion coefficient of a $100 \text{nm}$ nanocarrier was reported to be $2.4\times {10}^{-13} {\text{m}}^{2}/\text{s}$ [57], while this parameter decreased to $1.0\times {10}^{-14} {\text{m}}^{2}/\text{s}$ for the nanocarriers sized at $500 \text{nm}$ [58]. In the case of multistage nanocarriers which had size varied during the delivery process, experiments yielded a diffusion coefficient of $2.2\times {10}^{-12} {\text{m}}^{2}/\text{s}$ and $2.3\times {10}^{-11} {\text{m}}^{2}/\text{s}$ [59]. Moreover, the diffusion coefficient of $50 \text{nm}$ nanocarriers in the cellular microenvironment was reported as $7.7\times {10}^{-14} {\text{m}}^{2}/\text{s}$ [60]. Therefore, our study adopts values in a range from $1.0\times {10}^{-15} {\text{m}}^{2}/\text{s}$ to $1.0\times {10}^{-10}{\text{m}}^{2}/\text{s}$. The baseline value is set to be $1.0\times {10}^{-13} {\text{m}}^{2}/\text{s}$.

#### Microneedle properties

Microneedle length ($l$) precisely defines the penetrating depth of microneedles and the location where drugs are administrated. This geometrical property is usually hundreds of micrometres in practice. A $140\upmu{\text{m}}$ hollow microneedle was fabricated in Wang and colleagues’ work [61]; while the microneedles developed by Mukerjee et al. had their lengths located in the range of $250\sim 300 \upmu{\text{m}}$ [62]. Davis et al. applied a $500 \upmu{\text{m}}$ microneedle in their animal experiments on transdermal delivery of insulin [63]. In order to underscore its role in delivery outcomes, the following microneedle lengths are used in this study: a $100 \upmu{\text{m}}$ microneedle stops in the SC, while the lengths of $250 \upmu{\text{m}}$ and $400 \upmu{\text{m}}$ allow drugs to be infused into the PD. The $550 \upmu{\text{m}}$ and $700 \upmu{\text{m}}$ microneedles are sufficiently long to reach the RD. The baseline length is $250 \upmu{\text{m}}$.

Channel width ($d$) is another microneedle property that can be well controlled during fabrication. Mukerjee et al. reported a measurement of $10 \upmu{\text{m}}$ in their study [62]. In comparison, the microneedle fabricated by Yu et al. featured a channel width of $50 \upmu{\text{m}}$ [64], whereas Matteucci and colleagues constructed microneedles with a channel width of $84 \upmu{\text{m}}$ [65]. Hence, a range of $10\sim 90 \upmu{\text{m}}$ is adopted. This baseline width is $30 \upmu{\text{m}}$.

#### Infusion parameters

Infusion rate (${R}_{\text{in}}$) defines how fast the drugs are infused into the skin tissue. It should be carefully selected to not only guarantee effective delivery but also avoid possible tissue damage. The rate for a single microneedle was reported in the range of $0.05\sim 0.3 \upmu\text{L}/\text{min}$ [10, 66, 67]. The infusion into the skin through $3\times 3$ and $10\times 10$ microneedle arrays were at the overall rates of $1 \upmu\text{L}/\text{min}$ and $80 \upmu\text{L}/\text{min}$ [68]. Therefore, the infusion rate through a single microneedle was located in the range of $0.01 \upmu\text{L}/\text{min}$ to $8.89 \upmu\text{L}/\text{min}$. The flow rate was reported to be $83.99 \upmu\text{L}/\text{min}$ in the study on hollow microneedles for transdermal drug delivery systems against hemodynamic dysfunctions [69]. Therefore, the infusion rate ranges from $0.01 \upmu\text{L}/\text{min}$ to $100 \upmu\text{L}/\text{min}$ in this modelling study using a microneedle REV. The baseline infusion rate is $1.0 \upmu\text{L}/\text{min}$.

Infusion duration (${t}_{\text{in}}$) is the time window the drug administration through hollow microneedles is performed. Verbaan et al. reported a duration of 40 min [70], whereas Resnik et al. performed a 60-min infusion in their in vivo experiments on hollow microneedle-mediated transdermal delivery of insulin [68]. Because of these, a wide range of $1\sim 60 \text{min}$ is applied to cover these reported durations. The baseline infusion duration is $30\text{ min}$.

#### Environmental factors

Wind speed (air velocity, ${u}_{\text{air}}$) plays a critical role in determining the TEWL, thereby influencing ISF flow in skin tissues following the removal of microneedles. The Beaufort scale [71] is commonly used to judge the wind speed. Since the wind speeds of calm and gentle breezes are less than $0.45 \text{m}/\text{s}$ and $3.4\sim 5.4 \text{m}/\text{s}$, respectively, the range of $0.1\sim 5.0 \text{m}/\text{s}$ is employed to encompass daily life environments. The baseline wind speed is $0.1 \text{m}/\text{s}$ in this study.

Relative humidity ($RH$) quantifies the amount of water vapour present in a mixture of air and water in comparison to the maximum amount it could hold. This factor ranges from $0$ to $100 \%$ in this study, with a baseline value set to be $80 \%$.

### Boundary conditions

A constant infusion rate and drug concentration are imposed at the inlet of the microneedle CH, enabling drug delivery during the infusion process. The wall of the hollow microneedle is considered rigid with no fluid flux or drug flux during infusion. The microneedle removal after the administration makes the skin tissue at the cavity surface exposed to the atmosphere. Therefore, the zero gauge pressure and open boundary, defined in Eq. (21), are applied on this cavity surface for the transfer of ISF and drugs, respectively, enabling the loss of fluid and drugs to the environment.

21 $$\left\{\begin{array}{cc}-\mathbf{n}\cdot D\nabla C=0& \mathbf{n}\cdot {\mathbf{v}}_{\text{ISF}}\ge 0\\ C=0& \mathbf{n}\cdot {\mathbf{v}}_{\text{ISF}}<0\end{array}\right.$$

where $\mathbf{n}$ is the vector of the normal direction to the boundary, $D$ is the diffusion coefficient of nanocarriers or free drugs, and $C$ stands for the concentration. The surface of microneedle CH is a rigid wall, where the flow velocity and drug flux are zero.

TEWL is zero during the drug administration due to the coverage of the microneedle patch at the SC surface, preventing water loss to the environment. The flux of TEWL (${f}_{\text{TEWL}}$) after the removal of hollow microneedles is subject to the wind speed (${u}_{\text{air}}$) and relative humidity ($RH$) in the surrounding environment [27], as:

22 $${f}_{\text{TEWL}}={k}_{\text{g}}\frac{\left({a}_{\text{w}}-RH\right){p}_{\text{sat}}^{\text{o}}MW}{RT}$$

in which ${p}_{\text{sat}}^{\text{o}}=4.76 \text{kPa}$ refers to the water-saturated vapour pressure at the SC surface. The temperature at the skin surface is $32^\circ{\rm C}$, which is converted to an absolute temperature of $T=305.15 \text{K}$. The gas constant $R$ equals to $8.314 \text{J}/\text{mol}/\text{K}$. The molecular weight ($MW$) of water is $18 \text{g}/\text{mol}$. The mass transfer rate is ${k}_{\text{g}}=9.056\times {10}^{-3}{D}_{\text{air}}^{2/3}\sqrt{{u}_{\text{air}}/{L}_{\text{air}}}\left(\text{m}/\text{s}\right)$, in which ${D}_{\text{air}}=2.6\times {10}^{-5} {\text{m}}^{2}/\text{s}$ is the diffusivity of water in the air and ${L}_{\text{air}}=1.34\times {10}^{-1} \text{m}$ is the characteristic length [27]. The ambient relative humidity at the skin surface, ${a}_{\text{w}}$, could approximate relative humidity when dynamic equilibrium is arrived. An empirical equation can be then employed to describe the relation between the ${f}_{\text{TEWL}}$ and $RH$ [27], as:

23 $${f}_{\text{TEWL}}=-2.25\text{exp}\left(\frac{RH}{3.18}\right)-2.97\times {10}^{-3}\text{exp}\left(\frac{RH}{1.34\times {10}^{-1}}\right)-1.41\times {10}^{-15}\text{exp}\left(\frac{RH}{2.79\times {10}^{-2}}\right)+16.4 \left(\text{g}/{\text{m}}^{2}/\text{hr}\right)$$

Obeying conservation of mass, the flux of evaporated water at the surface of SC equates to the flux of water transporting from the VE to SC. Therefore, ${f}_{\text{TEWL}}$ is imposed at the SC-VE interface to consider TEWL after the removal of hollow microneedles. Because of the impermeable nature of SC to drugs, the local drug flux is always set to zero. The continuous boundary condition is imposed at the interfaces between different skin layers to enable ISF and drug transport [37, 72]. For the same reason, this boundary condition is also utilised at the interface between the CH and skin tissue during infusion. The fluxes of ISF and drugs in different forms are set to zero at the bottom of the computational domain [22]. The symmetric boundary condition and axis boundary condition are specified at the side of REV and the axis, respectively.

### Numerical methods

The governing equations are solved in COMSOL Multiphysics to generate numerical solutions. The time step is fixed at $0.001 \text{s}$ after a time-step sensitivity test. The sub-model for fluid transport is solved first under the no-infusion condition, where the infusion rate is zero, to obtain a steady-state solution to mimic the flow field before drug administration starts. This flow field is then imported to the fluid and drug transport models as the initial condition for transient simulations of transdermal drug delivery. The time for removing microneedles typically is much shorter compared to the 24-h simulation period. Therefore, the process of microneedle removal is neglected in this study. The treatment process is thereby divided into two stages: the infusion stage and the post-microneedle removal stage. The nanocarrier concentration in the microneedle CH is set as the infusate concentration at the onset of the administration, whereas the initial concentrations of free drugs and bound drugs are zero across the whole computational domain.

### Quantification of delivery outcomes

#### Spatially averaged concentration

The nanocarrier and released drug concentrations vary across different skin layers, subject to the microneedle-mediated infusion and the complex tissue-drug interplays shown in Fig. 1. Therefore, the spatially averaged concentration (${C}_{\text{avg}}$) is adopted to assess drug accumulation in every skin layer and blood, defined as:

24 $${C}_{\text{avg}}=\frac{\sum {C}_{\text{i}}{V}_{\text{i}}}{\sum {V}_{\text{i}}}=\frac{\sum {C}_{\text{i}}{V}_{\text{i}}}{V}$$

where ${C}_{\text{i}}$ is local drug concentration. ${V}_{\text{i}}$ is the local tissue volume. $V$ is the entire volume of the studied compartment.

#### Drug exposure over time

The efficiency can be accessed using drug exposure over time, calculated as the area under the curve ($AUC$) of the spatially averaged concentration of free drug against time, ${C}_{\text{F},\text{avg}}\left(t\right)$, as:

25 $$AUC={\int }_{0}^{t}{C}_{\text{F},\text{avg}}\left(\tau \right)d\tau$$

## Results

### Baseline drug delivery

**Fig. 3:**
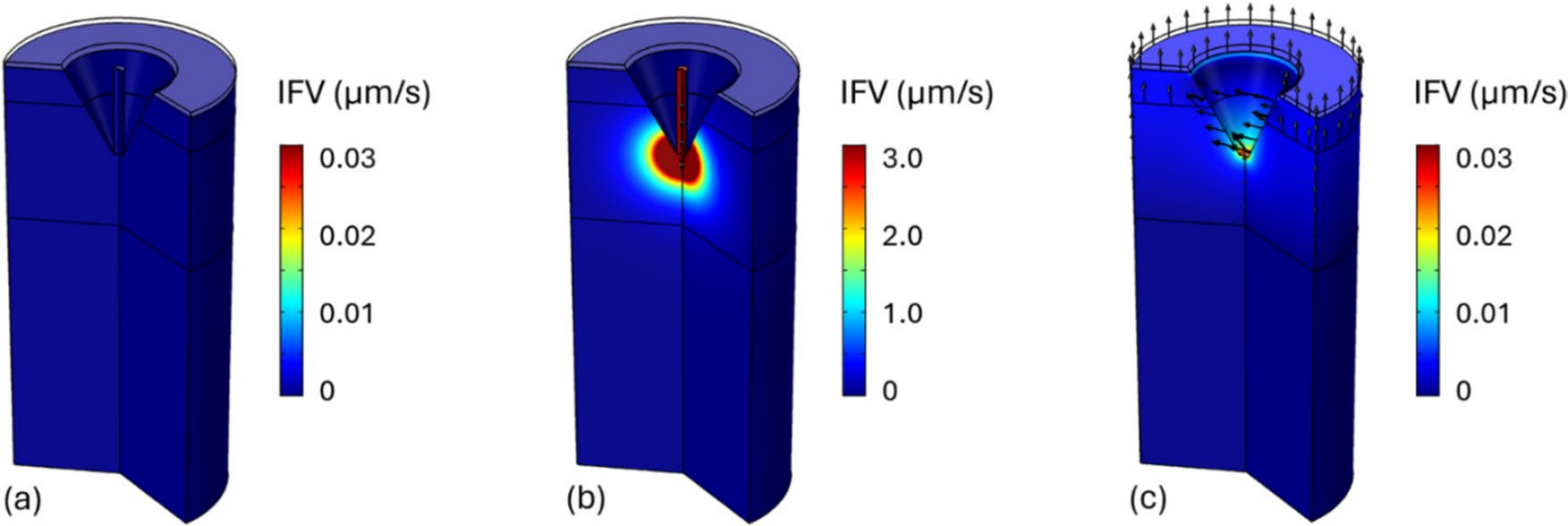
Infusate and interstitial fluid velocity (IFV) in the skin tissues before the infusion **a**, during the infusion **b**, and after the removal of the microneedle **c**. The flow direction is indicated by the black arrows. Baseline condition: $RH=80\%$, ${u}_{\text{air}}=0.1 \text{m}/\text{s}$, and ${R}_{\text{in}}=1\upmu\text{L}/\text{min}$

Nanocarriers are infused into the skin tissues via the hollow microneedle CH. The fluid transport model is solved throughout the entire domain to obtain the hydraulic environment of the nanocarrier and released drug movement. Figure 3 shows the fluid flow at different stages of the treatment. Before the infusion takes place, the fluid velocity remains at a significantly low level because the patch attached to the skin surface can prevent water from escaping into the atmosphere. In contrast, the flow can be greatly accelerated by the infusion, emanating from the microneedle CH towards the skin tissues. Within skin tissues, the ISF flow reaches peak velocity at the infusion site and gradually decelerates as it penetrates deeper. After the microneedle is removed, the skin tissues that were in contact with the microneedle are exposed to the air. Consequently, ISF can leave the tissue driven by the pressure difference between the tissue and the external environment. Moreover, water can also travel through the epidermis into the air since the patch no longer covers the skin surface. Unlike during infusion, the flow at this stage is from the deep layers of skin tissue to the surface, with velocities orders of magnitude lower.

**Fig. 4:**
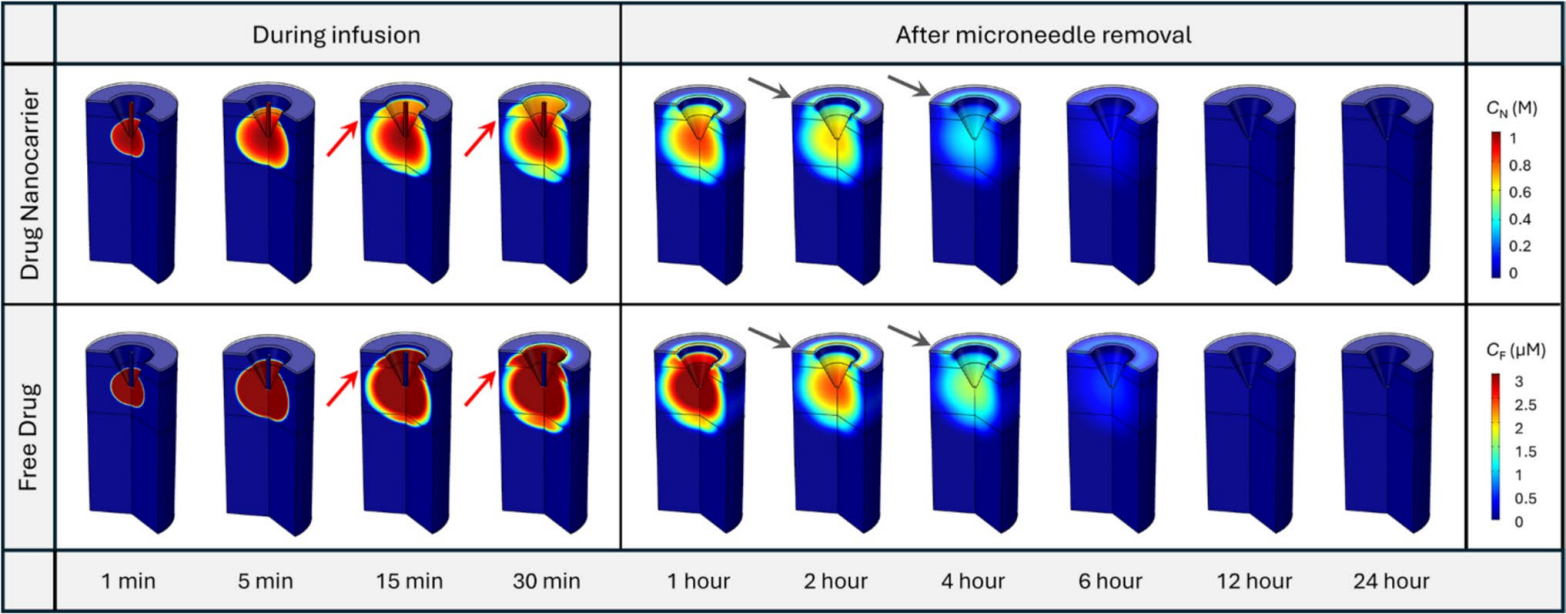
Spatial distribution of nanocarriers and free drugs at different time points in the skin in the baseline study. The upper panel displays the concentration distribution of nanocarriers, while the lower panel illustrates the concentration distribution of free drugs released from the nanocarriers

The spatial distribution of drug nanocarriers at different time points is shown in the upper panel of Fig. 4. The infusion through the hollow microneedle can successfully administrate nanocarriers into the PD where the infusion site is located. As the injection progresses, nanocarriers gradually move from the infusion site deeper into the PD, eventually permeating into the upper VE and the lower RD. The concentration front of nanocarriers exhibits a discontinuous pattern as they traverse between layers of skin tissue, particularly conspicuous at 15 min and 30 min, as highlighted by the red arrows. The travel of nanocarriers is notably slow within the PD. This is because while nanocarriers decrease due to drug release in all layers of the skin tissue, their loss is further exacerbated in the PD due to the effects of blood and lymphatic drainage. After the removal of the microneedle at 30 min, the concentration of nanocarriers throughout the entire skin tissue gradually decreases over time until it becomes invisible at 12 h. This can be attributed to drug release in the entire tissue and blood and lymphatic drainage at the PD. It is noteworthy that during this process, apart from the infusion site, the drug concentration at the SC-VE interface is also relatively high, as pointed out by the arrows in dark grey. This is due to the effect of TEWL which drives ISF to move towards the skin surface, as shown in Fig. 3(c). As a result, nanocarriers also travel from the deep tissue to the surface by convection and accumulate at this interface since the SC is impermeable to the nanocarriers. Similar distribution patterns can be found for free drugs shown in the lower panel, implying the direct impact of nanocarriers on free drugs. The concentration of free drugs in the microneedle CH remains low during infusion. This occurs because all drugs are encapsulated within the nanocarriers before entering the tissue and, importantly, the free drugs released within the tissue encounter difficulty in travelling back into the microneedle CH during the infusion process. These simulation results demonstrate the feasibility of delivering drugs to skin tissue through hollow microneedles.

**Fig. 5:**
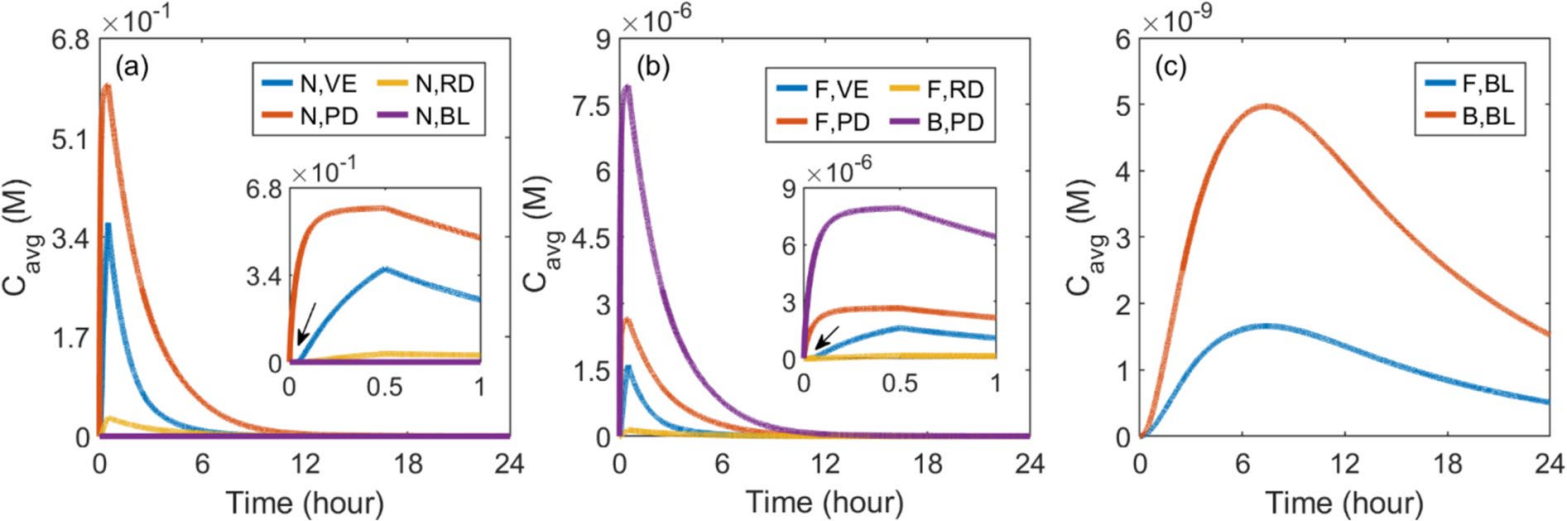
Drug concentrations in skin tissues and blood as a function of time. (**a**) The concentration of nanocarriers in skin and blood, and the concentration of released drugs in **b** skin and **c** blood

Figure 5(a) illustrates the nanocarrier concentration in skin tissues and BL as a function of time. Results show that this concentration continues to increase in all tissue compartments during the infusion and then gradually decreases to lower levels over time after the infusion ends. Given that the infusion site resides within the PD, as shown in Fig. 2, the concentration therein remains consistently high in the PD, followed by the VE and RD. Blood, the furthest end of this delivery process, exhibits the lowest concentration. Close observation of the first hour reveals an immediate spike in concentration within the PD once the infusion starts. Although similar trends exist in the VE and RD, there is a noticeable delay at the onset of infusion, as indicated by the arrow in black colour. As shown in Fig. 5(b), free drug concentrations present similar trends across skin layers and BL as the nanocarriers. However, it can be seen that most drug in the skin tissues exists in a form bound to protein. Its concentration is always maintained at three times the free drug concentration, determined by the drug-protein binding kinetics described by Eq. (15) and the drug properties given in Table 2. This also holds for concentrations in the BL, as shown in Fig. 5(c). Therefore, we will primarily focus on nanocarriers and free drugs in the subsequent parametrical studies, where an influencing factor is changed at a time to reveal its effect on the delivery results.

### Effect of nanocarrier properties

#### Release rate

**Fig. 6:**
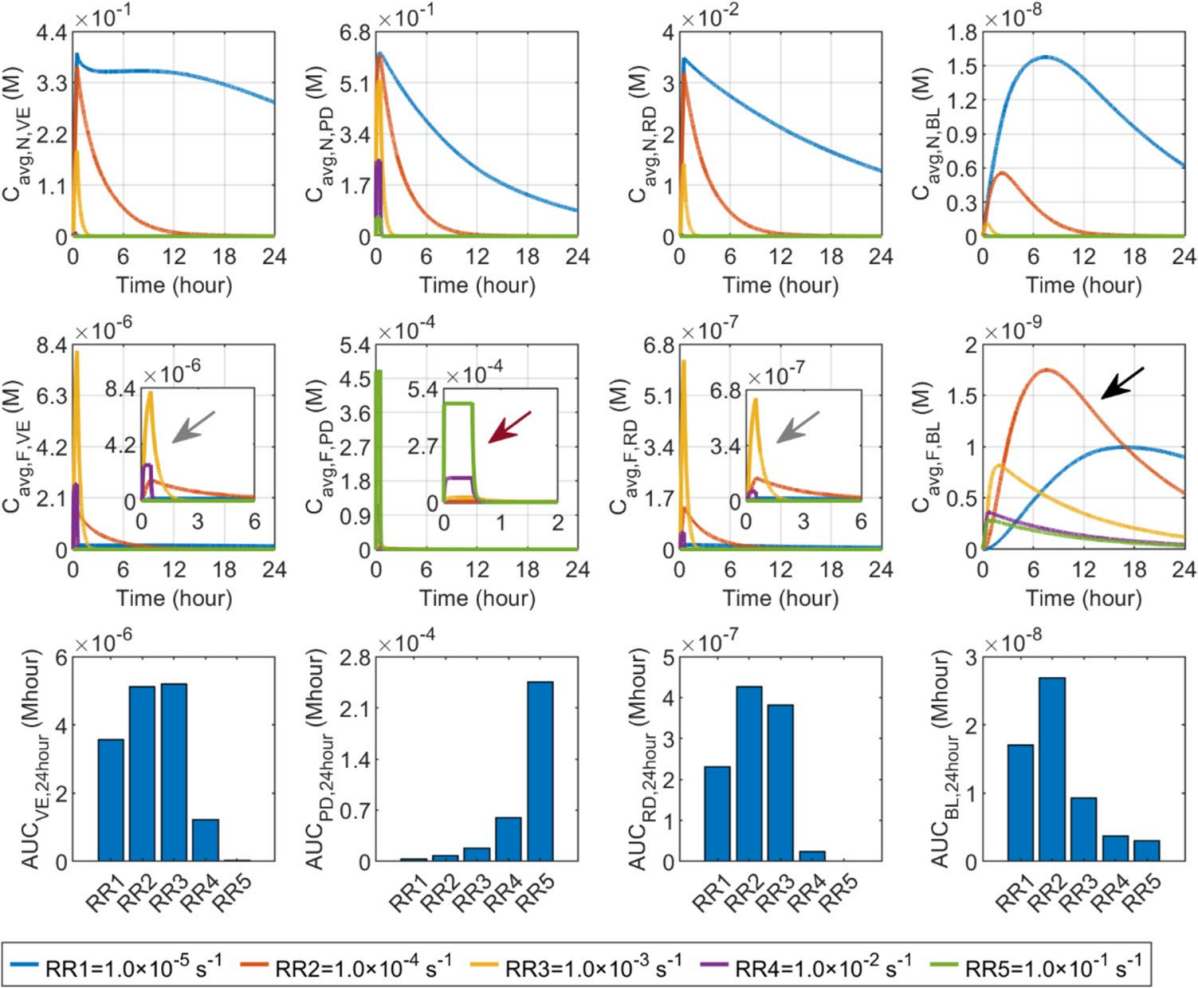
Effect of release rate (${k}_{\text{rel}}$) on transdermal delivery outcomes using hollow microneedles. Upper panel: time courses of nanocarrier concentration in skin layers and blood. Middle panel: time courses of free drug concentration in skin layers and blood. Lower panel: drug exposure over 24 h. The columns are, from left to right, VE, PD, RD and BL. This plot layout is also used in the following figures, in which the role of each factor is examined

The effect of release rate on delivery results in skin and BL is presented in Fig. 6. It can be found that accelerating drug release can effectively reduce nanocarrier concentrations in all skin layers and BL. In contrast, the responses of free drug concentration differ distinctly between compartments. Since nanocarriers are infused into the PD, a higher release rate allows drugs to be rapidly released, thereby quickly increasing the concentration of free drugs locally, as highlighted by the dark red arrow. Although a faster release also allows more free drugs to transfer from the PD to VE and RD, driven by the increased concentration gradient, it can also reduce the nanocarriers depositing in these two layers to release drugs locally. Therefore, the free drug concentration in the VE and RD are determined by the trade-off between these two terms, both influenced by the release rate. Modelling results show that the release rate of $1.0\times {10}^{-3} {\text{s}}^{-1}$ leads to the highest peak of free drug concentrations in these two layers, as pointed out by the grey arrows; However, they decrease faster as compared to the concentrations when drug release occurs at the rate of $1.0\times {10}^{-4} {\text{s}}^{-1}$. Similarly, the blood concentration of free drugs depends on the joint effects of local drug release from nanocarriers and the transfer of free drugs from the PD. The release rate of $1.0\times {10}^{-5} {\text{s}}^{-1}$ results in a more continuous drug supply over time, but the highest peak is achieved when drugs are released at the rate of $1.0\times {10}^{-4} {\text{s}}^{-1}$, as indicated by the black arrow. Drug exposure is determined by peak concentration and the change rate of concentration over time. The exposure in the PD can be significantly enhanced by raising the release rate, while this nanocarrier property should be optimised to maximise the drug exposure in the rest compartments. Specifically, the highest $AUC$ in the VE can be obtained when the releaser rate is $1.0\times {10}^{-3} {\text{s}}^{-1}$. $1.0\times {10}^{-4} {\text{s}}^{-1}$ is the optimum release rate for the BL and RD.

**Fig. 7:**
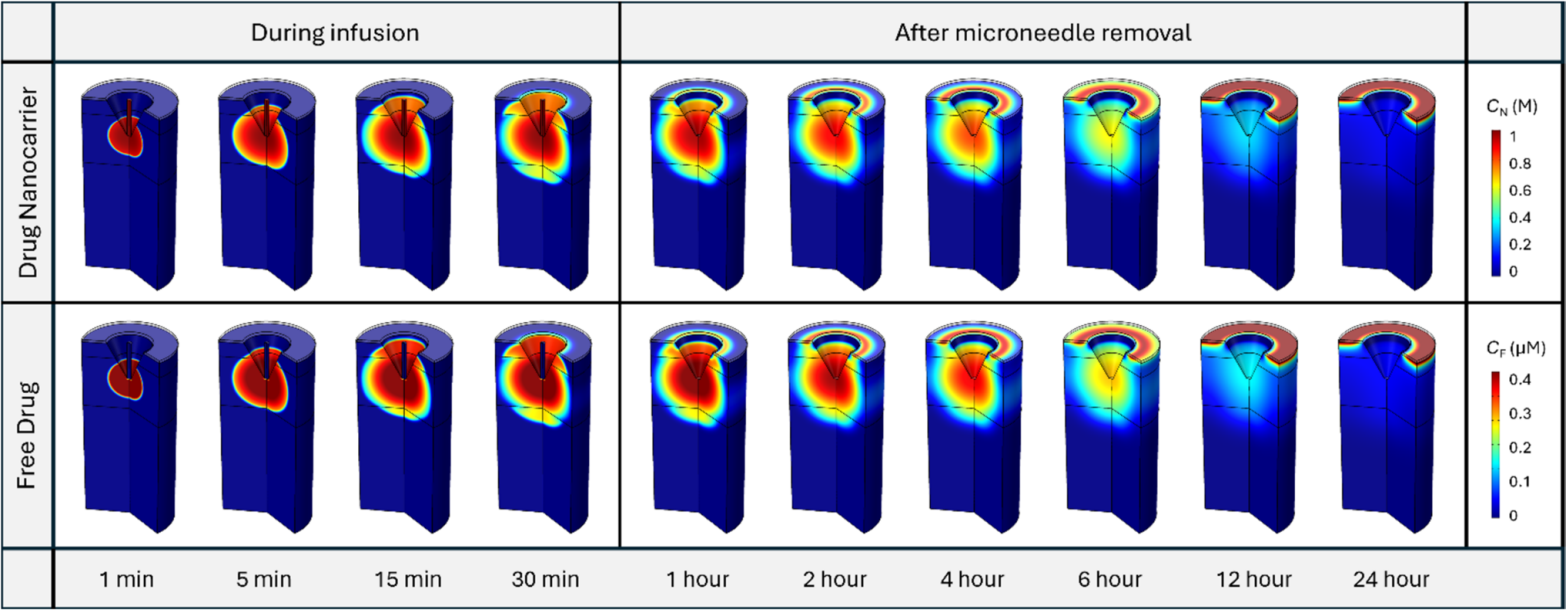
Spatial distribution of drug concentration when using nanocarriers with a release rate of $1.0\times {10}^{-5} {\text{s}}^{-1}$. The upper panel illustrates the concentration distributions of nanocarriers, while the lower panel depicts the distributions of free drug concentrations

An interesting finding in Fig. 6 is the nanocarrier concentration in the VE when the nanocarrier release rate is $1.0\times {10}^{-5} {\text{s}}^{-1}$. Unlike drug concentrations at other release rates that experience a sustained decrease after microneedle removal, this concentration first decreases rapidly after the removal, then levels off and gradually decreases over time. This trend can mainly be attributed to the complex drug transport processes in skin tissues. As the microneedle is initially removed, the concentration of nanocarriers experiences a rapid decline owing to drug release and dispersion into the environment through the cavity surface. However, as ISF migrates from deep tissues towards the skin surface, as shown in Fig. 3(c), nanocarriers from the PD travel into the VE by convection. This influx helps counterbalance the reduction in nanocarrier concentration due to release and loss to the environment, thereby maintaining a relatively stable average concentration of nanocarriers in the VE. Although nanocarriers can accumulate longer at the SC-VE interface due to the slower release rate, the nanocarrier concentration gradually decreases across the entire domain over time because of drug release, loss to the environment and blood and lymphatic circulating systems, as shown in Fig. 7. Consequently, the average concentration of nanocarriers in the VE reduces again with time proceeding.

#### Vascular permeability

**Fig. 8:**
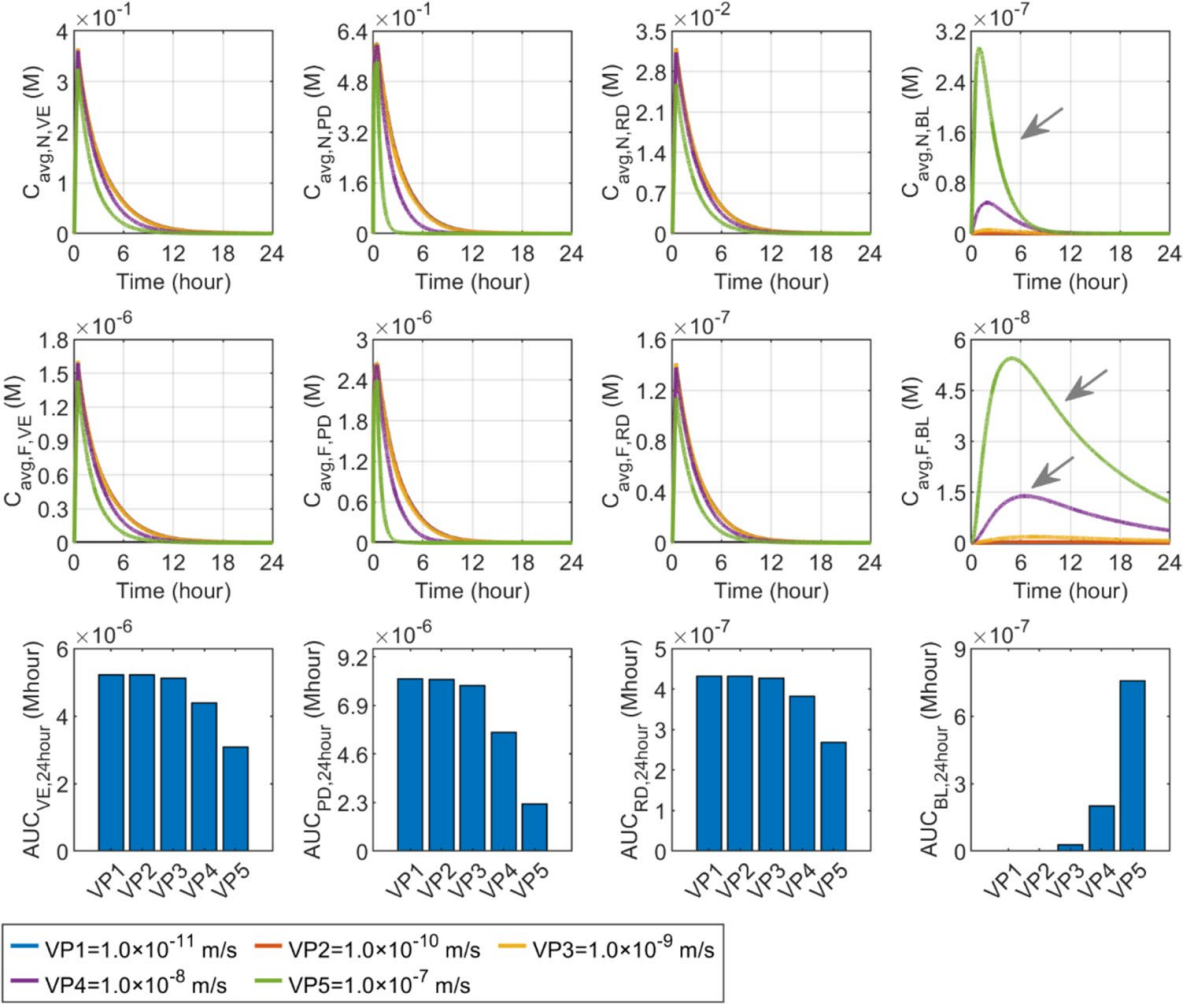
Effect of nanocarrier vascular permeability (${P}_{\text{N}}$) on transdermal delivery outcomes using hollow microneedles

Figure 8 shows the results of transdermal delivery in response to the changes in vascular permeability of nanocarriers. No significant effect can be found when the vascular permeability increases from $1.0\times {10}^{-11} \text{m}/\text{s}$ to $1.0\times {10}^{-9} \text{m}/\text{s}$. However, further raising this parameter can effectively enhance the transfer of nanocarriers into the BL, thereby reducing the amount of nanocarriers remaining in the PD and those able to travel to the VE and RD. Therefore, as vascular permeability increases from $1.0\times {10}^{-9} \text{m}/\text{s}$ to $1.0\times {10}^{-7} \text{m}/\text{s}$, nanocarrier concentrations can reach lower peaks and decline in a faster manner across all skin layers. Whereas, the concentration in the BL can be dramatically improved, as indicated by the grey arrows. Free drugs exhibit similar responses in each corresponding layer. Comparisons in the lower panel show that drug exposure in skin tissues reduces with the increased vascular permeability of nanocarriers, while the delivery outcomes in the BL can be improved.

#### Diffusion coefficient

**Fig. 9:**
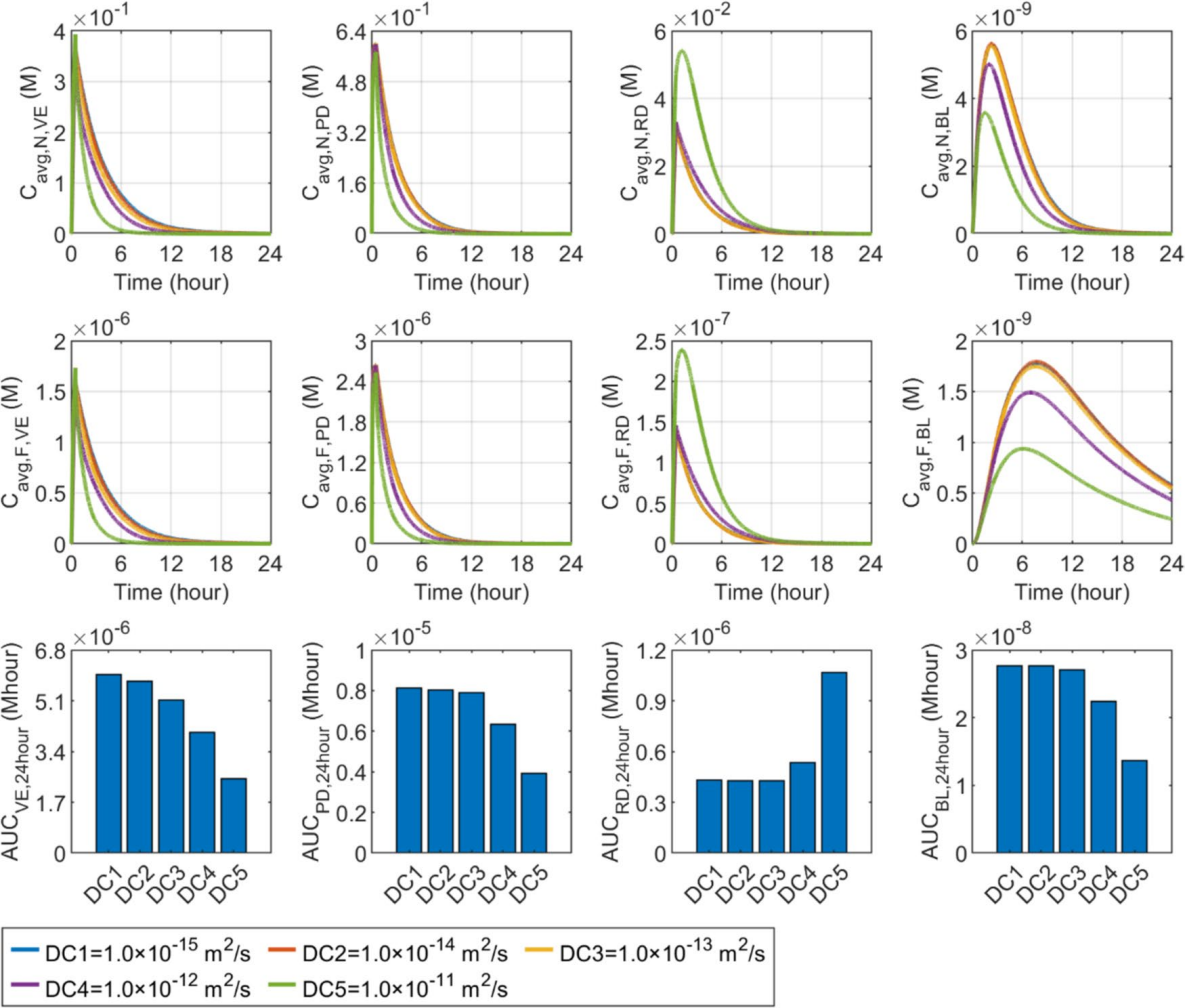
Effect of nanocarrier’s diffusion coefficient (${D}_{\text{N}}$) on transdermal delivery outcomes using hollow microneedles

Compared in Fig. 9 shows the effect of the nanocarrier diffusion coefficient on delivery outcomes in skin tissues and BL. Since nanocarriers are first infused into the PD, using nanocarriers with a higher diffusion coefficient is able to accelerate the movement of these nanocarriers into the downstream layers, leading to a more rapid decrease of the concentrations in the PD. This consequently reduces the nanocarriers that can cross the blood vessel and thereby lowers the concentration in the blood circulating system. On the contrary, the RD downstream of the delivery process can receive more nanocarriers. Therefore, its concentration presents a positive relation with the nanocarrier diffusion coefficient. While it is also possible that more nanocarriers enter the VE from the PD, enhanced diffusive transport would also result in more nanocarriers being lost to the environment at the cavity surface after microneedle removal. This can reduce the nanocarrier concentration in this skin layer. The free drug concentration exhibits a similar response as nanocarriers to the change in diffusion coefficient in each compartment. The lower panel shows that increasing the diffusion coefficient from $1.0\times {10}^{-15} {\text{m}}^{2}/\text{s}$ to $1.0\times {10}^{-13} {\text{m}}^{2}/\text{s}$ has a less significant effect on drug exposure in the skin and BL. Further increase can improve the treatment in the RD, while the delivery to the PD, VE and BL are reduced simultaneously.

### Effect of microneedle properties

#### Microneedle length

**Fig. 10:**
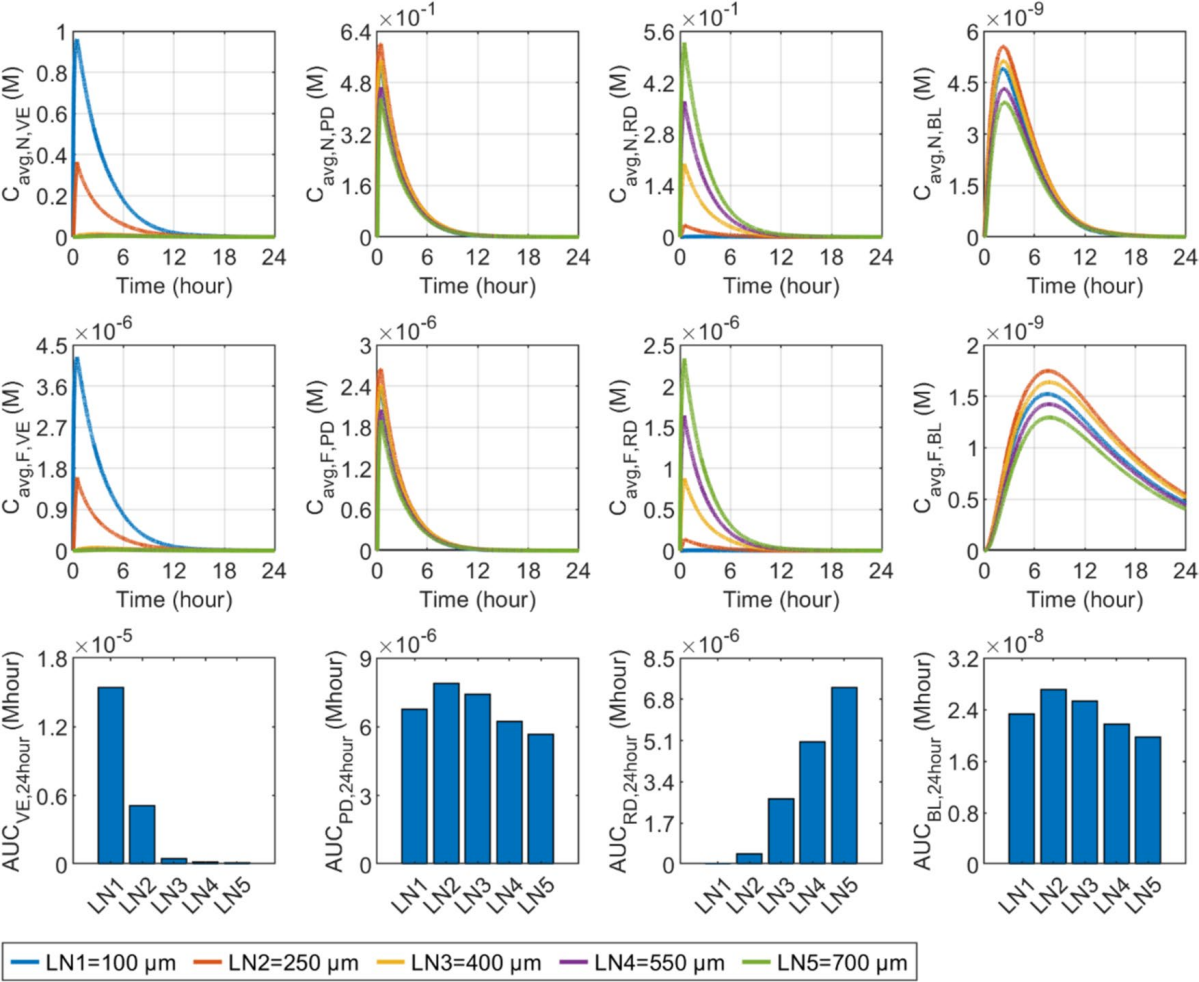
Effect of microneedle length ($l$) on transdermal delivery outcomes using hollow microneedles

Figure 10 shows the delivery results in response to changes in microneedle length. Nanocarrier concentrations in VE can be substantially enhanced through the utilisation of $100 \upmu\text{m}$ microneedles, as the microneedle is designed to penetrate solely the SC for drug delivery to this specific layer. However, the concentrations in this layer are dramatically reduced using longer microneedles. While the $100 \upmu\text{m}$ microneedle can indeed transport nanocarriers to the PD, a greater concentration in this layer is attained with $250 \upmu\text{m}$ microneedles, which are capable of reaching this depth. Simulations further show that the concentrations in the PD reduce with the increase in the microneedle length. Since blood vessels mainly exist in the PD, similar trends can be found for the nanocarrier concentrations in the BL. To be different, longer microneedles lead to increased concentrations of nanocarriers in the RD. This is attributed to their capability to penetrate the superficial layers effectively, facilitating the delivery of nanocarriers to this deepest skin layer. Free drugs present a similar response, as shown in the middle panel. Analyses of the $AUC$ show that drug exposure in the VE decreases rapidly with the length of the microneedle. In contrast, longer microneedles result in greater exposure to drugs in the RD. There is an optimal microneedle length for the PD and BL.

#### Channel width

**Fig. 11:**
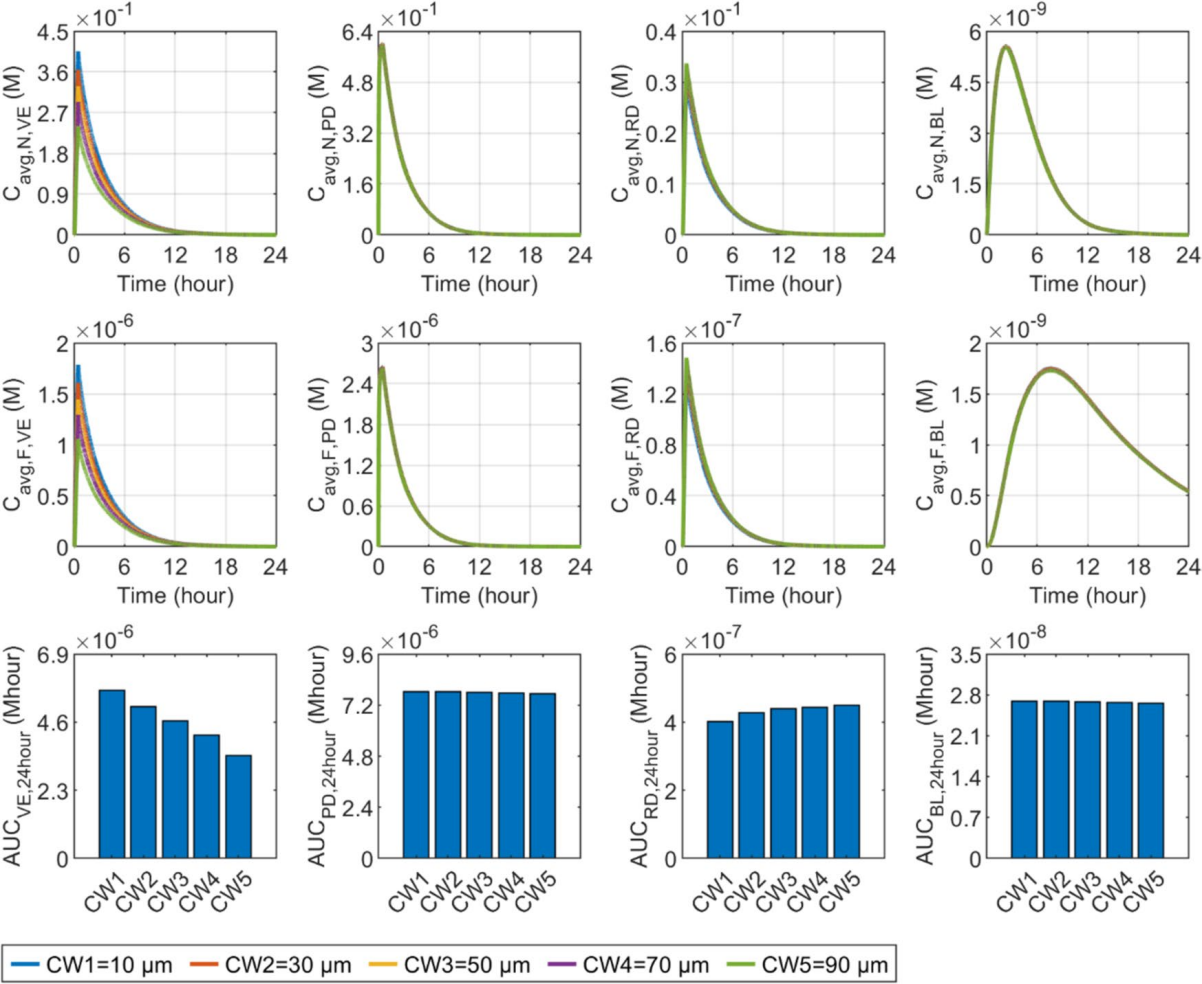
Effect of channel width ($d$) on transdermal delivery outcomes using hollow microneedles

The effect of channel width on drug availability in each skin layer and BL is displayed in Fig. 11. It is worth noting that the infusion rate is kept identical in all simulations, where only the channel width varies. The use of a narrow channel can effectively accelerate the ISF flow that enhances the nanocarrier travel into the VE by convection, allowing the concentration to achieve a higher peak at the end of infusion. However, the effect on the concentration in the PD is modest. Since the infusion rate remains constant across simulations, the flux of nanocarriers infused into the PD does not change visibly with the channel width. The findings also indicate that decreasing the channel width may lead to a slight reduction in the concentration within the RD. Free drugs exhibit similar sensitivity to channel width, as shown in the middle panel. The lower panel denotes that drug exposure in the VE is inversely correlated with channel width, however, widening the channel can marginally improve delivery to the RD. The outcomes in the PD and BL are insensitive to this factor.

### Effect of infusion settings

#### Infusion rate

**Fig. 12:**
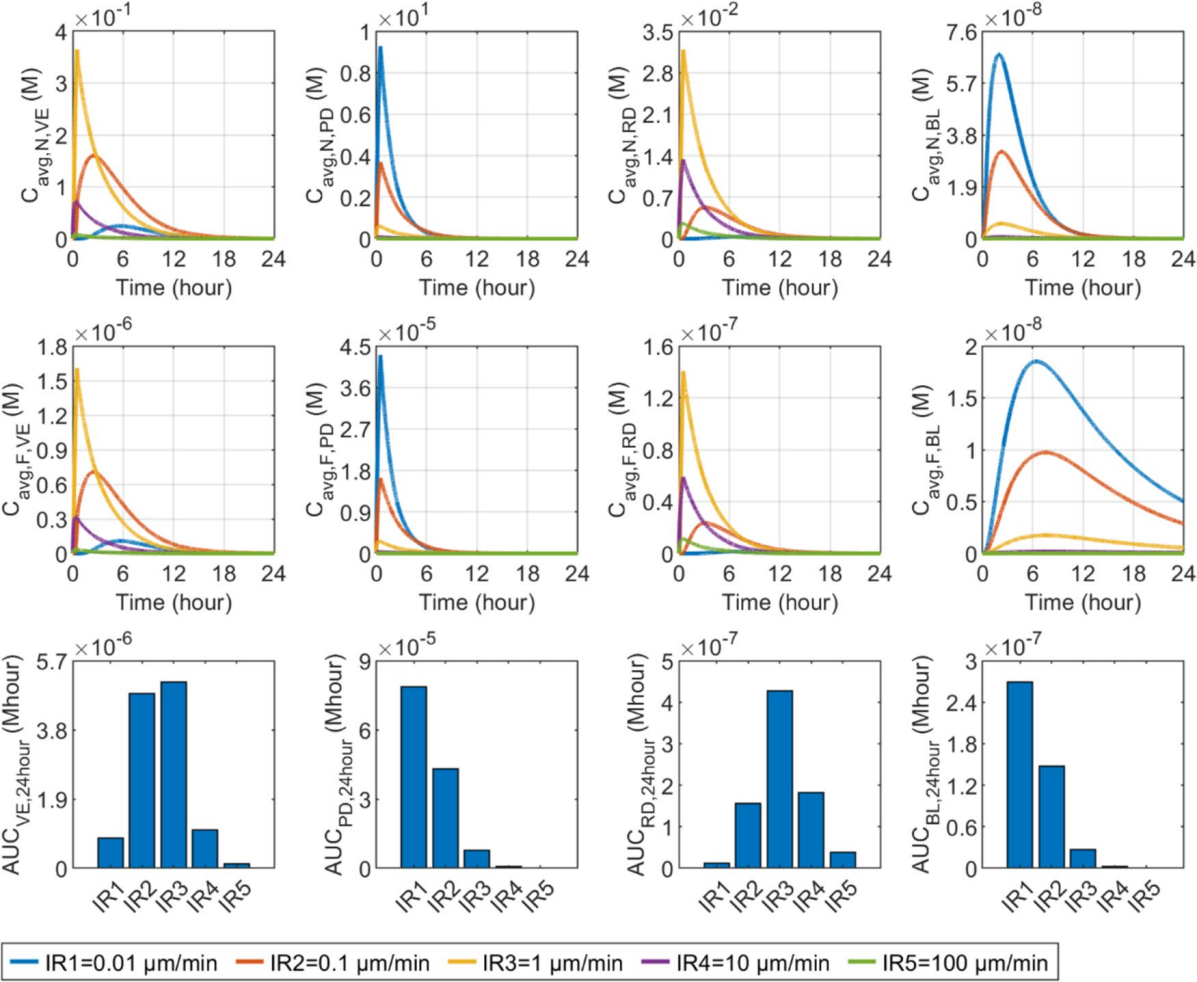
Effect of infusion rate (${R}_{\text{in}}$) on transdermal delivery outcomes using hollow microneedles

Figure 12 compares the delivery outcomes of hollow microneedle-mediated transdermal drug delivery at varying infusion rates. The infusate concentration is changed accordingly to maintain the total dose and infusion duration the same in all the simulations. A slower infusion with higher infusate concentration can successfully increase the concentrations in the PD, as this effectively reduces drug movement by convection into the VE and RD. As a result, drug concentrations in these two downstream layers decrease. On the other hand, while faster infusions hold the potential to augment drug transfer by convection into the VE and RD, the significance of drug diffusion diminishes due to the reduced concentration gradient resulting from a lower infusate concentration. This may lead to lower concentrations in these two skin layers. Results show that the highest concentrations in the VE and RD occur when the infusion rate is $1 \upmu\text{L}/\text{min}$. The drug concentrations in the BL present similar sensitivity as the PD due to the distribution of blood capillaries. The lower panel shows that exposure to drugs in the PD and BL increases when the infusion rate decreases, while $1 \upmu\text{L}/\text{min}$ is the optimum for maximising the delivery outcomes in the VE and RD.

#### Infusion duration

**Fig. 13:**
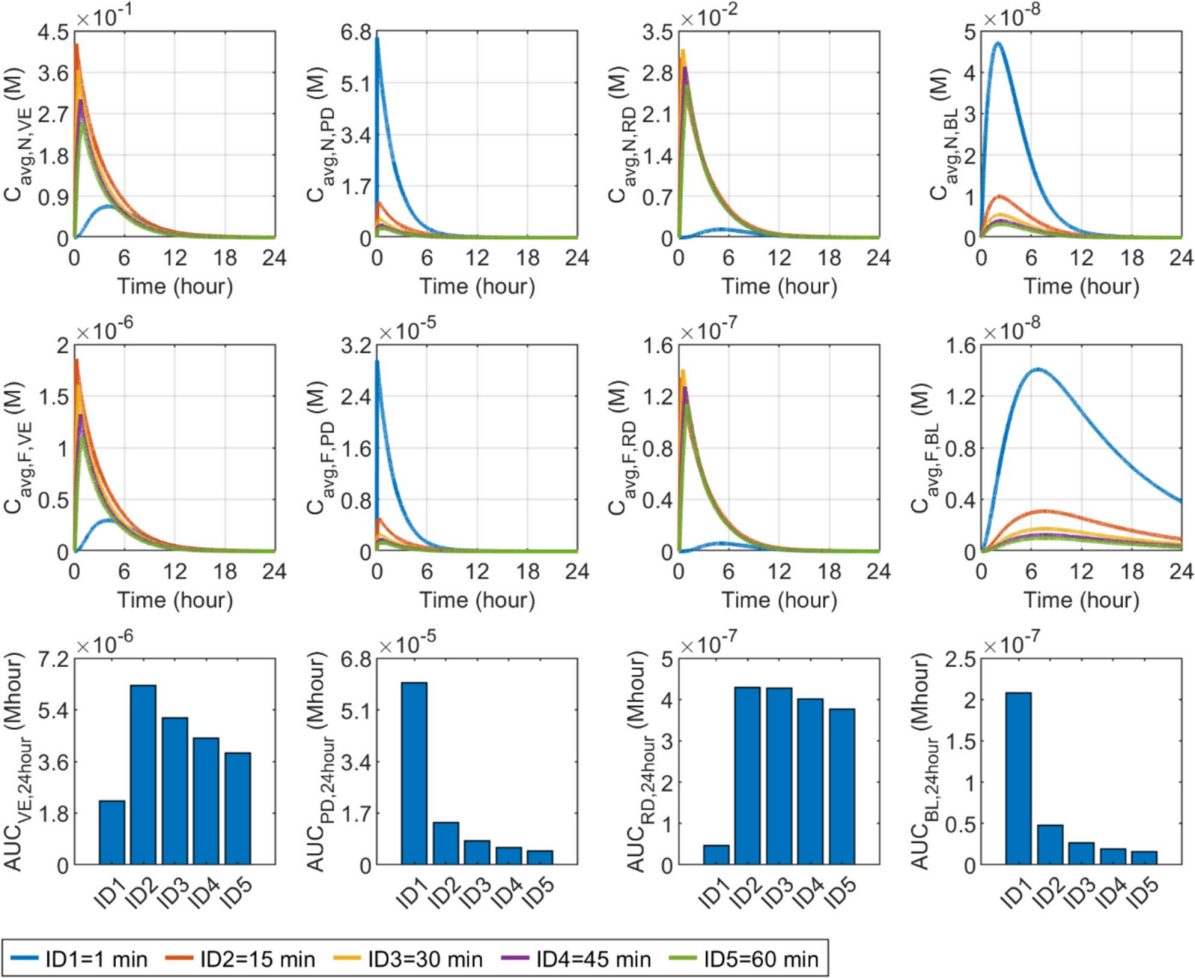
Effect of infusion duration (${t}_{\text{in}}$) on transdermal delivery outcomes using hollow microneedles

Figure 13 displays the influence of infusion duration on the delivery outcomes in skin tissues and BL. The infusate concentration is changed accordingly to maintain the total dose and infusion rate identical in all simulations. The highest peak of nanocarrier concentration in the PD is achieved when the infusion is completed within 1 min. Since all the nanocarriers are delivered in such a small time window, the concentration can be sharply increased. Simultaneously, the availability of nanocarriers is significantly reduced in the rest skin layers, leading to the lowest concentrations. Prolonging infusion duration allows more nanocarriers to travel from the PD to VE and RD, thereby increasing the nanocarrier concentrations in these two layers. However, it should be noted that during prolonged infusion, the infusate concentration needs to be reduced accordingly to maintain the same total dose and infusion rate. This reduction further decreases the concentration gradient from the infusion site to the deep skin tissues, thereby slowing down the spread of the nanocarriers through diffusion and therefore lowering the concentration. Results show that the nanocarrier concentration reaches its peak when the duration is 15 min and 30 min in the VE and RD, respectively. Similar responses are seen in free drug concentrations in all skin layers, due to the direct impact of nanocarriers. Comparisons in the lower panel indicate the most effective delivery in the PD and BL occurs when the infusion duration is short. Whereas, the optimal infusion duration is 15 min and 15 ~ 30 min for the VE and RD, respectively.

### Effect of environmental factors

#### Wind speed

**Fig. 14:**
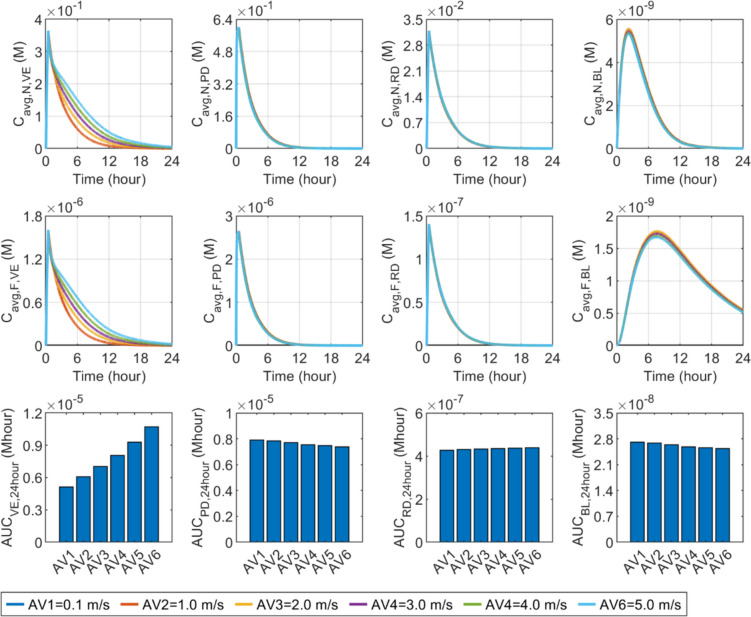
Effect of wind speed (${u}_{\text{air}}$) on transdermal delivery outcomes using hollow microneedles

Figure 14 compares the effect of wind speed on the delivery results in the skin and BL. The same nanocarrier and free drug concentrations can be found respectively at the infusion stage in all simulations. This is because the microneedle patch completely covers the surface of the skin, preventing the skin from coming into contact with air. After microneedle removal, higher wind speed can significantly boost TEWL, thus hastening the flow of ISF to the skin surface. This facilitates a greater drug passage from the deeper skin layers into the VE, consequently retarding the decline in drug concentration within this layer. Conversely, a marginal decrease in peak drug concentrations occurs in the PD and BL. As depicted in the lower panel, drug exposure in the VE improves with wind speed. Nevertheless, wind speed exhibits a minimal effect on drug delivery to deeper skin tissues, underscoring its influence as primarily confined to the superficial skin tissues.

#### Relative humidity

**Fig. 15:**
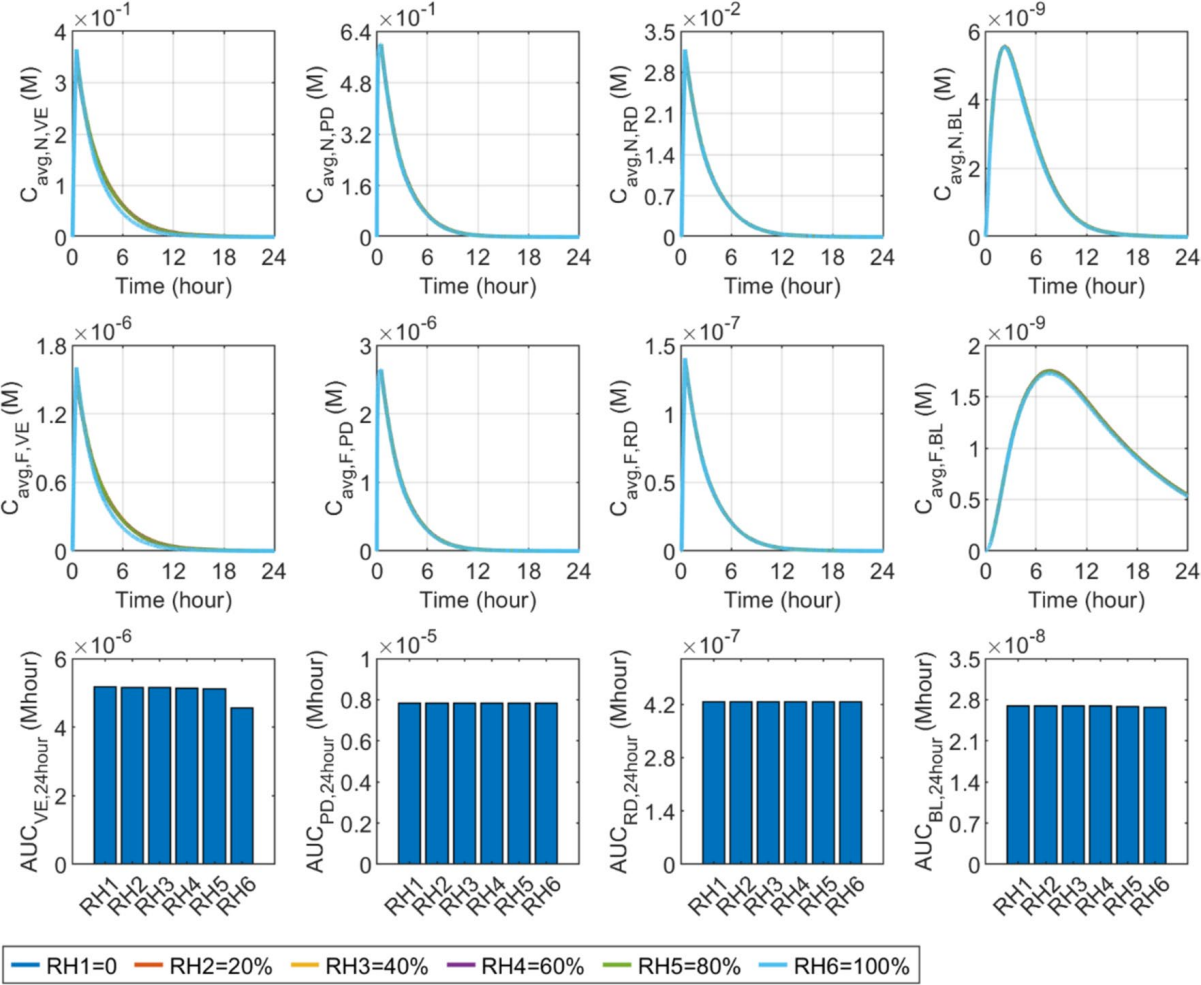
Effect of relative humidity ($RH$) on transdermal delivery outcomes using hollow microneedles

Similarly to wind speed, changes in relative humidity are capable of influencing the delivery after the removal of the microneedle patch. The sensitivity of delivery outcomes to relative humidity is shown in Fig. 15. Higher relative humidity can inhibit the evaporation of water at the skin surface, thereby reducing TEWL. This consequently slows down the transfer of ISF and drugs from the deeper skin layers to the VE. The results show that the relative humidity varying from 0 to 80% has a limited impact on drug availability in the skin and BL. Further raising this parameter can reduce the concentrations and drug exposure in the VE. The effect of relative humidity on the delivery results in the rest skin tissues and BL is insignificant.

## Discussion

Hollow microneedles can successfully deliver drugs into the skin tissues and BL by piercing the SC, which is impermeable to most external substances. Compared to conventional transdermal delivery in which drugs are directly applied to the SC surface, drug availability can be significantly improved using hollow microneedles. In particular, ISF flow plays an important role. On the one hand, direct infusion can accelerate this flow and thereby improve drug penetration into deep skin tissue via convection. On the other hand, ISF moves towards the skin surface due to TEWL after the microneedle is removed. This flow can entrain the drug to move to the superficial skin tissue and accumulate under the SC.

Drug distribution using hollow microneedles is highly non-uniform in the skin tissues, making this drug delivery method potentially conducive to achieving local and precise drug delivery. Our results demonstrate significant variations in delivery outcomes among different skin layers in response to various influencing factors. For the PD where the infusion site is located, drug availability and exposure can be effectively enhanced by increasing the release rate and reducing the infusion rate or infusion duration. However, separate optimisation is needed for these factors when targeting the VE and RD. The use of nanocarriers that can diffuse faster in skin tissues would be beneficial to improve delivery to the RD while reducing drug availability in the remaining tissue compartments. Among the studied factors, increasing vascular permeability is the only way to enhance the drug systemic availability without increasing drug concentration in any skin layer. This finding suggests the possibility of using transdermal drug delivery to treat non-dermal diseases. As a factor controllable during manufacturing, selecting the microneedle with the desired length to position the infusion site in the target skin layer would be the most direct approach to enhance the local delivery outcomes. Narrowing the infusion channel can only improve the delivery to the VE, while drug depositions in the PD and BL are less influenced. Furthermore, environmental factors also need to be considered, although they primarily affect the delivery outcomes in the VE. Specifically, exposure to an environment with high wind speed or low relative humidity can enhance TEWL, thereby improving the drug delivery to the VE. In summary, thoughtful design of these factors and their combination based on the target delivery site can enhance therapeutic efficacy while minimising possible negative effects of drugs on normal tissues.

Nano-sized drug carriers have been extensively studied and are now employed in practice due to their advantages in controlled drug release [73–75]. A range of their properties, including those analysed in this study, can be engineered through formulation and fabrication techniques [73]. In the context of transdermal delivery using hollow microneedles, the selection of nanocarriers plays a crucial role in determining delivery outcomes. Modelling results show that nanocarriers with higher vascular permeability can significantly enhance drug concentrations in the blood. This finding has important implications for drugs targeting the circulating system or other tissues, e.g. insulin. Such efficient transport across vessel walls can be achieved through surface modifications with specific ligands [55, 76]. For deeper skin tissue targeting, particularly in applications such as cancer treatment, nanocarriers must exhibit high diffusivity. This property can be achieved by reducing the dimension of drug nanocarriers or enhancing their zeta potential [77–80]. Moreover, nanocarriers can be generally classified based on their release mechanisms, which are intrinsically linked to their formulation [81]; these include diffusion-controlled release, as seen in poly(ethylene-co-vinyl acetate) nanocarriers; erosion or degradation-controlled release, exemplified by poly(ε-caprolactone) nanocarriers; swelling-controlled release, as observed in hydrogel-based nanocarriers; and burst release, characteristic of lipid-based nanocarriers. Aside from the formulation, the release rate is further influenced by factors such as pH value [82, 83], temperature [84], and the properties of the encapsulated drug [81]. The layer-specific response of the skin to drug release rates, as demonstrated in this study, offers a reference for selecting nanocarriers with optimised release profiles for targeted transdermal delivery.

Owing to its minimally invasive nature and user-friendly administration, transdermal delivery through hollow microneedles has emerged as a promising strategy for large-scale applications such as vaccination [85], as well as for repeated and long-term treatment, including chronic disease management and hormone therapy [86]. In vivo experiments have confirmed its superiority to traditional subcutaneous injection in improving drug delivery effects [11, 87]. This can help reduce both the frequency of administration and the required dose. Our study further underscores that through the careful selection and optimisation of the drug delivery system properties with respect to the specific disease and delivery requirement, it is possible to achieve localised and controlled delivery with high drug concentrations in the target regions and minimised drug accumulation in normal tissues. These target regions may include specific skin layers or systemic circulation. This not only enhances therapeutic efficacy but also reduces side effects. However, it is worth noting that this study focuses solely on the drug concentration and distribution. For specific diseases or drugs, the current model requires further development to incorporate their unique drug transport mechanisms, if any, and pharmacodynamics to enable disease-specific or drug-specific investigations. For instance, the combination of the delivery model of insulin with glucose kinetics can be used to explore the application of microneedles in diabetes therapy.

**Fig. 16:**
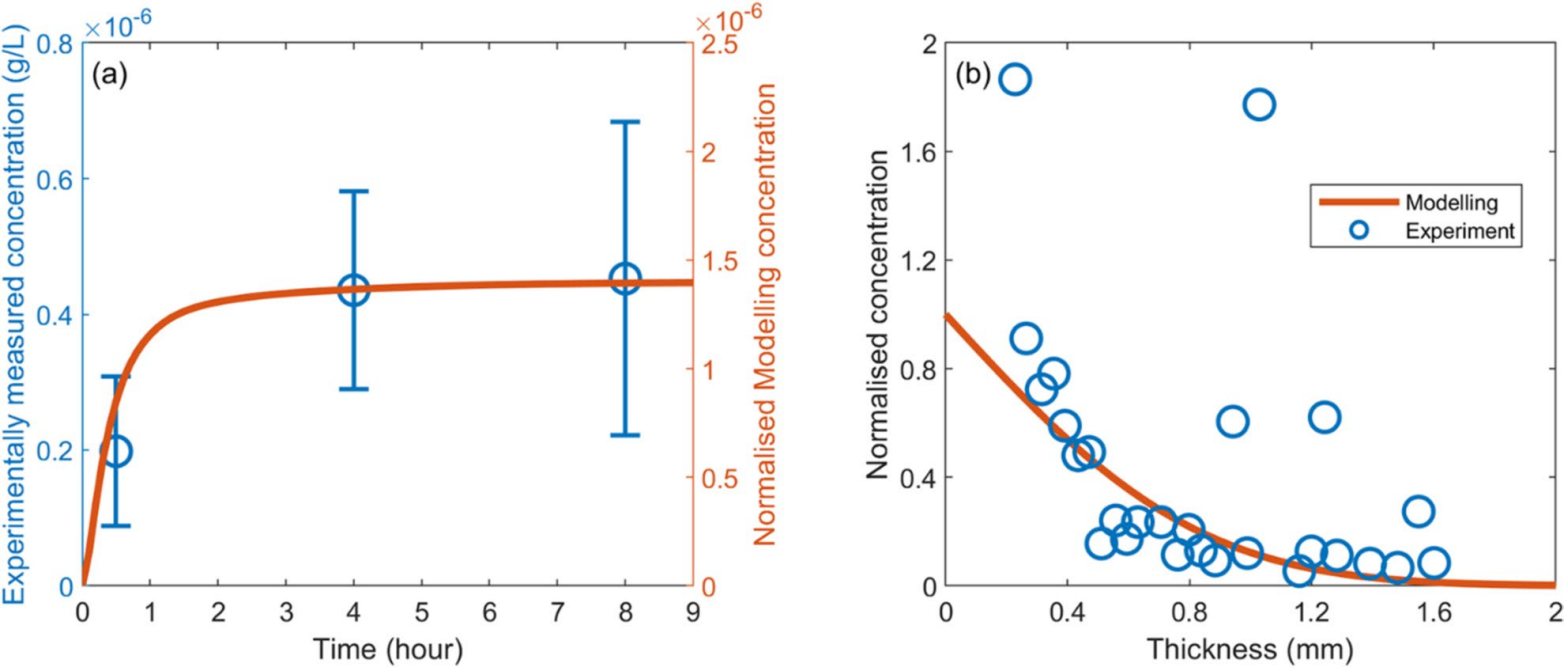
Model Validation. (**a**) Comparison of the model-predicted time course of insulin plasma concentration with experimental measurements [63]. The modelling was conducted to simulate transdermal delivery using the same hollow microneedle array and under identical conditions to those employed in animal experiments [63]. (**b**) Comparisons of the modelling predicted distance courses of triamcinolone acetonide concentration in skin tissue with experimental measurements [23]. The model parameters and experimental results [23] are obtained from the literature

The mathematical model employed in this study is developed to describe the key delivery processes in transdermal drug delivery using hollow microneedles. The modelling predicted concentration of insulin in the plasma is compared with the measurements from animal experiments using rats [63] in Fig. 16(a). Specifically, the elimination rates of insulin in plasma and skin tissues are $2.22\times {10}^{-3} {\text{s}}^{-1}$ and $4.83\times {10}^{-5} {\text{s}}^{-1}$, respectively, while its vascular permeability is $4.17\times {10}^{-8} {\text{s}}^{-1}$ [88]. The diffusivity (${\text{m}}^{2}/\text{s}$) of insulin in tissues can be estimated by $$\begin{array}{cc}D=1.778\times {10}^{-8}{\left(MW\right)}^{-0.75}& \left(32<MW<69000\right)\end{array}$$ [89]. Due to the molecular weight of insulin is $MW=5808 \text{g}/\text{mol}$ [90], its diffusivity is calculated as $2.67\times {10}^{-11} {\text{m}}^{2}/\text{s}$. The blood volume of rats is reported to be $64\text{ mL}/\text{kg}$ [91]. The good agreement in Fig. 16(a) suggests that the modelling can well predict the variation trend of drug concentration, rather than the exact concentration values. Furthermore, the model is applied to simulate triamcinolone acetonide transport in the dermis, using the reported drug transport properties [23]. The predicted concentrations as a function of penetrating depth are plotted against the experimental measurements [23] in Fig. 16(b), showing good consistency. Similar comparisons were also reported in the previous modelling studies on topical and transdermal drug delivery [23, 25] and drug delivery to other tissues [92]. However, it is crucial to note that mathematical modelling is generally limited to predicting delivery outcomes qualitatively. This is because, on the one hand, a mathematical model that can accurately describe all drug transport processes remains lacking. Most current models are developed to catch the major processes, whereas the less dominant processes are either simplified or ignored. On the other hand, simulating drug delivery requires a substantial quantity of model parameters reflecting the properties of drug delivery systems and tissues. Obtaining a full set of these parameters is still challenging. As an alternative, representative values of the parameters from different sources are usually applied. An example is the collection of parameters used in the model validation study on insulin, as explained above. Despite these, qualitative predictions can still be used in cross-comparisons to identify the effect of influencing factors to improve this transdermal drug delivery system. To further improve accuracy, the mathematical modelling needs to be more closely integrated with experimental research. For instance, experimental studies on physiological and biophysical-chemical processes can help develop and refine mathematical models for more detailed stages of drug delivery. *In vivo* experiments can also provide valuable measurements of tissue parameters.

Doxorubicin is selected as the representative drug for this study due to its established use in transdermal drug delivery [33, 34] and its well-characterised transport properties, as listed in Table 2. In contrast to other microneedle types that predominantly rely on diffusion for drug delivery into tissues, hollow microneedles infuse drugs directly into the skin. This thereby makes convection-driven transport, facilitated by enhanced interstitial fluid flow, a more significant mechanism. It should be noted that transport properties vary between drugs. A single drug is used since this study focuses on the impact of microneedle and nanocarrier properties, clinical parameters, and environmental factors. However, our previous studies on drug infusion into other tissues have demonstrated that the responses of drugs to the infusion-enhanced interstitial fluid flow [93] and changes in the tissue microenvironment [94] heavily depend on their transport properties. Therefore, drug-specific studies can be performed in the future to benefit the development of a specific drug delivery system or delivery strategy.

Microneedle insertion is accompanied by tissue damage, leading to compression of the surrounding tissue, which in turn reduces tissue porosity and permeability. This reduction in tissue permeability is likely to be non-uniform, depending on the geometric features of the microneedles (e.g. shape, size, and length) and the arrangement of microneedles on the supporting patch (e.g. tip-to-tip distance). However, there is a lack of studies that report the degree of tissue compression at each location in the different skin layers, which have varying mechanical properties. More importantly, the mathematical relationship between the compression degree and the change in permeability of different skin layers remains unclear. Therefore, the current model focuses on drug transport without accounting for the microneedle insertion profile. To address this, experiments are required to measure the mechanical properties of each skin layer, such as tissue permeability and poroelasticity, under varying levels of compression. These data can then be used to enhance the model by incorporating a tissue mechanics module to simulate the effects of microneedle insertion on drug transport.

**Fig. 17:**
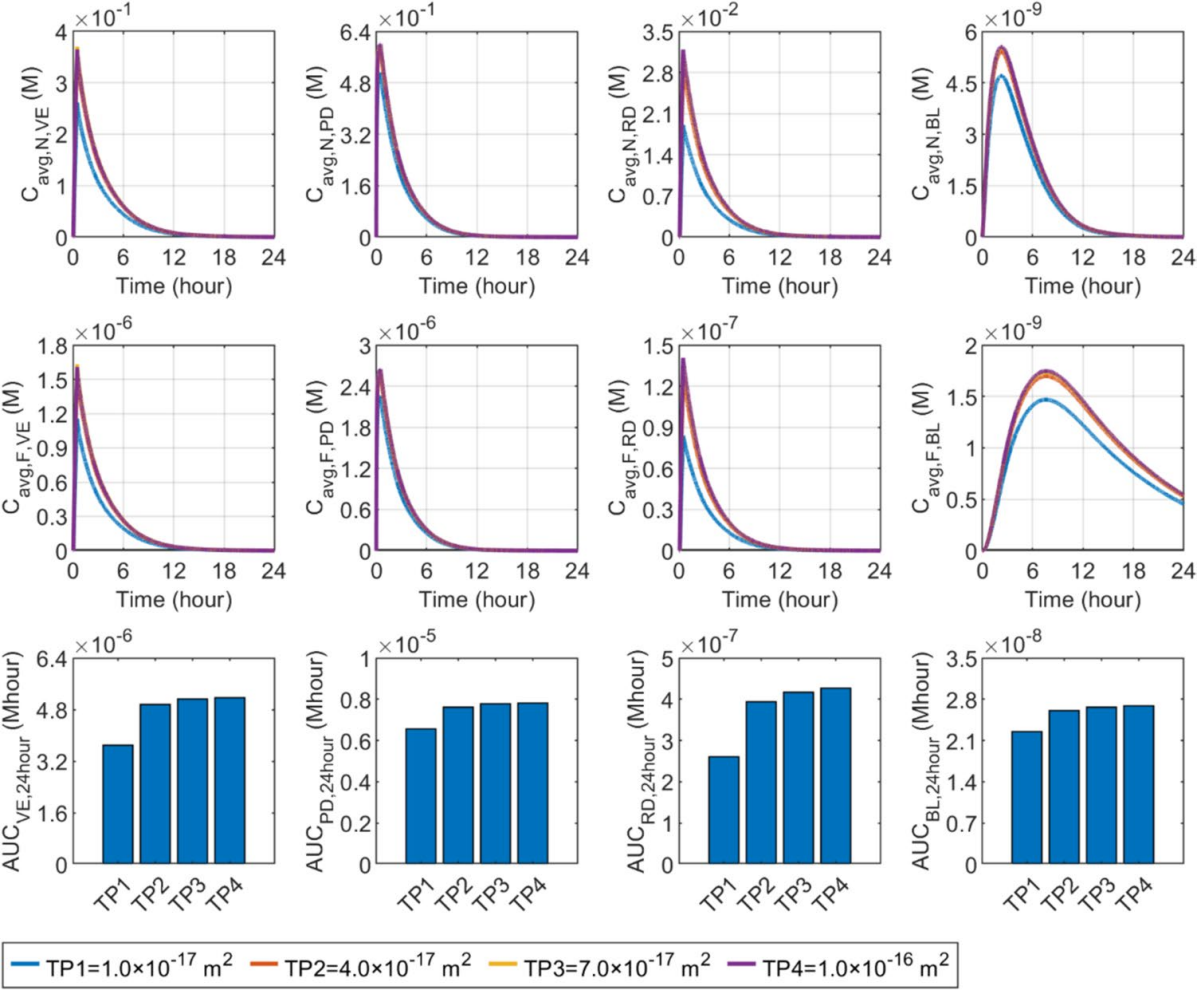
Effect of the changed tissue permeability during drug infusion on the delivery outcomes. The original tissue permeability (TP4) is uniformly reduced to 10% (TP1), 40% (TP2), and 70% (TP3) in the first 30 min when the infusion takes place. The tissue permeability restores to its original after the microneedle removal

The experiments demonstrated that the permeability of collagen-aminoglycan gel can be reduced to as low as $10\%$ when the compression degree changes from $0$ to $40\%$ [95]. Therefore, a parametric study discusses the effect of permeability reductions of $10\%$, $40\%$, and $70\%$ of the original value on drug delivery outcomes. Due to the lack of the map of compression degree, we assume that the change in tissue permeability of the skin during infusion is uniform. The modelling prediction results are summarised in Fig. 17. The results show that drug concentration decreases with decreased tissue permeability. This is because less permeable tissue reduces the interstitial fluid flow, thereby weakening the convective transport of drugs. However, the effect of changes in tissue permeability on drug delivery results is nonlinear. Only when tissue permeability decreases to $40\%$ of its original value or less does drug availability decrease significantly. It should be noted that assuming uniform changes in tissue permeability in skin tissue may lead to inaccurate estimates of its impact. A more precise analysis requires understanding the degree of tissue compression at each location and its mathematical relationship to tissue permeability, which are currently unclear. It is also worth pointing out that as tissue permeability decreases, drug concentrations in various layers of the skin and the blood change monotonically. This indicates that the above simulations that do not consider changes in tissue permeability can only provide qualitative predictions of drug delivery outcomes to determine the role of different influencing factors.

This study also includes other limitations and assumptions. (1) This study focuses on ISF flow and drug transport in the skin tissues. Higher infusion rates have the possibility to significantly increase pressure on the skin tissue, thus leading to tissue deformation. This may cause changes in tissue properties. Since the infusion rates in this study are selected in the reported experimental range [10, 66–69], designed to avoid significant tissue deformation in practice, such a complex process is not included. Future studies can further develop the model by employing fluid–solid interaction to consider the impact of pressure-induced tissue deformation. (2) The cone-shaped microneedle with a central cylindrical infusion channel [31, 32] is selected as a representative to perform affordable computational simulations in the parametrical studies. The geometry of microneedles can vary markedly depending on the particular design [13, 96]. The infusion channel can also be designed with different geometrical features and allocated at the side to avoid possible blockage by tissues during microneedle insertion. Particularly, the location and geometry of the infusion channel are believed to play a pivotal role in determining the outcomes of hollow microneedle-mediated transdermal drug delivery. The mathematical model developed in this study is still valid to simulate the transdermal delivery using microneedles with different shapes and geometrical properties. However, it is important to note that their geometries may no longer be considered 2D axisymmetric. Therefore, the 3D geometries of these microneedles are required. The increase in the complexity of the computational domain and the number of mesh elements would demand additional computational resources, raising computational expenses. A separate study focused on the impact of geometric features of hollow microneedles can benefit the design for improving delivery outcomes. (3) The current model is developed based on the principles of mass transfer and fluid mechanics to focus on drug delivery after microneedle implantation. Hence, simulating the insertion process and profile is out of scope. To account for these, the model needs to be further developed to integrate a tissue mechanics module that can simulate tissue damage and deformation [97]. In addition, an interface is needed to connect the tissue mechanics module with the existing fluid flow and drug delivery modules to consider the effects of tissue deformation on the transport properties of drug and skin tissues. (4) Temperature is another important environmental factor that can influence drug delivery in the skin. Particularly, arteriovenous anastomoses can close when the temperature of skin tissue is below the lower end of the thermoneutral zone [98]. This disconnection between small arteries and veins can consequently cause changes in ISF flow and drug transport in the skin and to the BL. However, as a result of the lack of an accurate mathematical model to describe this process, the role of temperature is not examined here. Results from future physiological studies can be used to develop such a model and combined with the flow and drug transport model in this study to determine the effect of temperature.

## Conclusions

Transdermal drug delivery using hollow microneedles has been studied under various delivery conditions. Modelling results demonstrate that delivery outcomes are significantly influenced by ISF flow, which is determined by infusion during administration and TEWL after microneedle removal. Drug availability and exposure vary greatly among skin layers and BL, depending on various factors such as nanocarrier properties, microneedle properties, clinical settings, and environmental factors. Specifically, drug concentrations in the PD, where the infusion site is located, increase with the drug release rate, but are inversely related to the infusion rate and infusion duration. However, each of these three factors should be optimised individually to improve delivery to the VE and RD. The use of nanocarriers with high diffusion coefficients or vascular permeability can increase drug concentrations in the RD or BL, respectively, while reducing delivery outcomes in other compartments. Microneedle length also needs to be carefully selected to locate the infusion site directly at the target layer to increase local drug concentration. Wider infusion channels result in lower drug concentrations in the VE but can cause a marginal increase in drug availability in the RD. However, this microneedle geometrical property has a limited impact on the PD and BL. Delivery to the VE is positively correlated with the environmental factors that can accelerate TEWL, while drug availabilities in the PD, RD, and BL are not sensitive to such factors. These findings can be leveraged to improve transdermal drug delivery through the utilisation of hollow microneedles.

## Data Availability

The datasets generated during and/or analysed during the current study are available from the corresponding author on reasonable request.

## Abbreviations

B: Bound Drug to Proteins
BL: Blood
CH: Hollow Channel
F: Free Drug
IFS: Infusate
ISF: Interstitial Fluid
LY: Lymph
MN: Microneedle
N: Nanoparticle-Encapsulated Drug
PD: Papillary Dermis
RD: Reticular Dermis
REV: Represented Elementary Volume
SC: Stratum Corneum
TEWL: Trans-Epidermal Water Loss
TIS: Skin Tissue
VE: Viable Epidermis

## Symbols

*AUC*: Area under the curve
*d*: Half of channel width
*D*: Diffusion coefficient
*C*: Concentration
${C}_{\text{avg}}$: Volume-averaged concentration
${C}_{\text{i}}$: Local drug concentration
*d*: Channel width
${k}_{\text{clr}}$: Plasma clearance rate
${k}_{\text{d}}$: Physical degradation rate
${k}_{\text{pb}}$: Rate of drug association with proteins
${k}_{\text{pu}}$: Rate of drug dissociation from proteins
${k}_{\text{rel}}$: Drug release rate
*l*: Microneedle length
***n***: Normal direction to the boundary
*N*: Number of microneedles in an array
*P*: Vascular permeability
*r*: Microneedle base radius
$${R}_{\text{in}}$$: Relative humidity
$$RH$$: Infusion rate
*s*: Half of the tip-to-tip distance
*t*: Time
$${t}_{\text{in}}$$: Infusion duration
${u}_{\text{air}}$: Wind speed, air velocity
$${V}_{\text{dis}}$$: Distribution volume
${V}_{\text{i}}$: Local tissue volume
${v}_{\text{m}}$: Michaelis-Menten kinetics constant
${V}_{\text{max}}$: Michaelis-Menten kinetics constant

## Greek letter

$$\kappa$$: Tissue permeability
$$\mu$$: Viscosity
$$\pi$$: Osmotic pressure
$$\rho$$: Density
$\sigma$: Osmotic reflection coefficient
